# Treatment of Drug-Induced Liver Injury

**DOI:** 10.3390/biomedicines11010015

**Published:** 2022-12-21

**Authors:** Rolf Teschke

**Affiliations:** 1 Department of Internal Medicine II, Division of Gastroenterology and Hepatology, Klinikum Hanau, D-63450 Hanau, Germany; rolf.teschke@gmx.de; Tel.: +49-6181-21859; Fax: +49-6181-2964211; 2 Academic Teaching Hospital of the Medical Faculty, Goethe University Frankfurt/Main, D-60590 Frankfurt am Main, Germany

**Keywords:** DILI, DILI treatment, ferroptosis, ROS, RCTs, updated RUCAM, N-acetyl-p-aminophenol (APAP), N-acetylcysteine (NAC), antioxidants, clausenamide, glucocorticoids, iron chelators, polyene phosphatidylcholine, probiotics, S-adenosyl-methionine, silymarin, ursodeoxycholic acid (UDCA)

## Abstract

Current pharmacotherapy options of drug-induced liver injury (DILI) remain under discussion and are now evaluated in this analysis. Needless to say, the use of the offending drug must be stopped as soon as DILI is suspected. Normal dosed drugs may cause idiosyncratic DILI, and drugs taken in overdose commonly lead to intrinsic DILI. Empirically used but not substantiated regarding efficiency by randomized controlled trials (RCTs) is the intravenous antidote treatment with N-acetylcysteine (NAC) in patients with intrinsic DILI by N-acetyl-p-aminophenol (APAP) overdose. Good data recommending pharmacotherapy in idiosyncratic DILI caused by hundreds of different drugs are lacking. Indeed, a recent analysis revealed that just eight RCTs have been published, and in only two out of eight trials were DILI cases evaluated for causality by the worldwide used Roussel Uclaf Causality Assessment Method (RUCAM), representing overall a significant methodology flaw, as results of DILI RCTs lacking RUCAM are misleading since many DILI cases are known to be attributable erroneously to nondrug alternative causes. In line with these major shortcomings and mostly based on anecdotal reports, glucocorticoids (GCs) and other immuno-suppressants may be given empirically in carefully selected patients with idiosyncratic DILI exhibiting autoimmune features or caused by immune checkpoint inhibitors (ICIs), while some patients with cholestatic DILI may benefit from ursodeoxycholic acid use; in other patients with drug-induced hepatic sinusoidal obstruction syndrome (HSOS) and coagulopathy risks, the indication for anticoagulants should be considered. In view of many other mechanistic factors such as the hepatic microsomal cytochrome P450 with a generation of reactive oxygen species (ROS), ferroptosis with toxicity of intracellular iron, and modification of the gut microbiome, additional therapy options may be available in the future. In summation, stopping the offending drug is still the first line of therapy for most instances of acute DILI, while various therapies are applied empirically and not based on good data from RCTs awaiting further trials using the updated RUCAM that asks for strict exclusion and inclusion details like liver injury criteria and provides valid causality rankings of probable and highly probable grades.

## 1. Introduction

Drug-induced liver injury (DILI) remains an important concern among scientists, physicians, regulators, and experts in the field, with a focus on general aspects [1,2,3,4,5,6] or more specifically on drugs such as multiple antidepressants [7], teriflunomide [8], tigecyc-line [9], or potassium para-aminobenzoate [10]. The last three decades have witnessed substantial efforts on the question of how best to ensure the diagnosis of DILI in patients with abnormal liver tests (LTs) under treatment with drugs. Efforts started in 1993 with an international consensus meeting of DILI experts, who created the Roussel Uclaf Causality Assessment Method (RUCAM) [11,12], updated in 2016 [13]. In retrospect, RUCAM followed principles of artificial intelligence (AI), aiming to clarify difficult issues by simplifying complex processes and providing structured algorithms with specific elements to be individually scored [14,15]. As a consequence of its broad appreciation, RUCAM was used in 81,856 DILI cases worldwide that were published from 1993 up to the middle of 2020 [16]. RUCAM was also applied in 996 DILI cases found among patients with COVID-19 infections [17], published in 2020 and 2021 in several reports [18,19,20,21,22,23], with 72 additional RUCAM-based DILI published so far in 2022 [24] and a listing of various suspected drugs [17,18,19,20,21,22,23,24].

The importance of RUCAM for assessing causality in DILI cases was highlighted in review articles published by the group of Lewis et al. [25,26,27,28], the Chinese Society of Hepatology (CSH) together with the Chinese Medical Association (CMA) in their guidelines for the diagnosis and treatment of DILI [29], the DILI consensus guidelines of the Asia Pacific Association of Study of Liver (APASL) [30], another international consensus conference [31], and European DILI registries as critically summarized [32]. LiverTox does not fulfil the requirement of a professional causality assessment [33,34,35], nor is this goal achieved by other attempts such as electronic variations published in the past [36] or recently [37], as they all lack method validation [36,37] and were applied by the inventors only [36]. It is common knowledge that DILI often is not DILI [33,34,35,38], as cases can be explained by alternative causes [38]. Reporting DILI features should be based on DILI cases assessed using RUCAM, as shown in many published case reports [16] and briefly summarized recently [39]. RUCAM was also among the topics in a recent scientometric study by independent DILI experts from China not affiliated with any known DILI circle [40]. This analysis was highly appreciated among DILI experts [15].

In this article, therapeutic options for patients with DILI are considered, a particular challenge that requires a step-by-step approach. First, molecular and mechanistic considerations are discussed as background information for why specific drugs were used to treat DILI. Second, study protocols have to be analyzed regarding quality and fulfilling criteria of randomized controlled trials (RCTs). Third, key questions must be answered whether or not DILI cases were assessed properly using a robust diagnostic algorithm of causality assessment like the updated RUCAM to verify the DILI diagnosis. Prevention modalities using pharmaceuticals for potentially upcoming liver injury during drug treatment are outside of this analysis.

## 2. Literature Search and Source

The PubMed database and Google Scholar were searched for articles by using the following key terms: drug-induced liver injury (DILI) and treatment, and treatment efficacy, and clinical course, and RUCAM. Limited to the English language with a few exemptions, publications from each search terms were analyzed for suitability of this review article. The publication search was complemented from the large private archive of the author. The final compilation consisted of original papers, case reports, consensus reports, and review articles with the most relevant publications included in the reference list of this review.

## 3. Definitions

### 3.1. Idiosyncratic vs. Intrinsic DILI

By convention, DILI is caused by chemical drugs leading to idiosyncratic or intrinsic liver injury [13,15]. Idiosyncratic liver injury is due to the interaction between the drug used in recommended daily doses and a susceptible individual [15], whereby this type of injury can be caused by virtually any conventional, regulatory approved drug [13,16]. This is as opposed to intrinsic liver injury, which is due to a drug overdose, with paracetamol, also known as acetaminophen or N-Acetyl-p-aminophenol (APAP), as a typical example [15]. Consequently, patients with idiosyncratic DILI used their drugs commonly for some days, weeks, or months, while in the context of an acute intoxication, the drug intake is mostly limited to one or two days [13,15].

### 3.2. Real Liver Injury Versus Liver Adaptation or Tolerance

To firmly analyze the efficacy of drug treatment in DILI patients, a correct classification of DILI is essential using liver test (LTs) abnormalities of serum alanine aminotransferase (ALT) activities ≥ five times of the upper limit of normal (ULN) and/or alkaline phosphatase (ALP) activities ≥ two times of the ULN [13]. Chemical drugs may lead not only to classical liver injury but also to liver adaptation, also known as tolerance, defined as ALT activities < five times of the ULN or ALP < two times of the ULN [15]. In the context of drug efficacy studies in DILI patients, DILI study cohorts occasionally also included patients with liver adaptation, which leads to incorrect results of drug efficacy. The differences between the liver adaptation and real liver injury are listed with additional details of clinical features (Table 1).

Reports on DILI must consider essential diagnostic criteria to classify them as real liver injury cases and not as cases of liver adaptation. Abbreviations: ALT, alanine aminotransferase; ALP, alkaline phosphatase; DILI, drug-induced liver injury; ULN, upper limit of the normal range. This table is derived from a previous open-access journal [15].

### 3.3. Liver Injury Pattern

Study protocols conceptualized for drug treatment in DILI were often based on a special type of liver injury pattern also known as a phenotype, not relying on liver histology obtained through invasive liver biopsy but rather on analysis of serum LTs, considering the ratio R, obtained by using multiples of ULN of ALT and ALP to be divided as ALT: ALP [13]. The liver injury is hepatocellular if R ≥ 5, the liver injury is cholestatic if R ≤ 2, and the liver injury is mixed if 2 < R < 5 [13,14,15]. These three categories are found in most DILI cases [16] but they may not necessarily reflect drug-induced microvesicular steatosis hepatitis due to amiodarone, as an example [41]; drug-induced hepatic sinusoidal obstruction syndrome (HSOS) caused by oxaliplatin [42]; or drug-induced autoimmune-like hepatitis (DIAIH) [43].

### 3.4. RUCAM

RUCAM is a structured, transparent, and user-friendly causality assessment method validated by positive re-exposure tests of DILI cases as a gold standard. Serving as an objective and quantitative diagnostic algorithm [11,12,13], RUCAM is privileged to provide a robust causality assessment for drugs with suspected implication in DILI [13]. It outperforms, in terms of numbers of published RUCAM-based DILI cases [16], any other method [44] including all electronic RUCAM modifications, a futile attempt of some that are classified as gamechangers with respect to the well-functioning and worldwide-accepted RUCAM as outlined recently [36,37]. RUCAM is based on seven distinct domains comprising key elements that are well defined and provide individual scores [13]. Among the RUCAM domains of the hepatocellular injury, for instance, are the time to onset from the beginning (or the cessation) of the drug/herb use (scores +2 or +1), course of ALT after cessation of the drug/herb (scores +3 to −2), risk factors (scores +1 or 0), concomitant drug(s) and herb(s) (scores 0 to −3), search for alternative causes (scores +2 to −3), knowledge of product hepatotoxicity (scores +2 to 0), and response to unintentional re-exposure (scores +3 to −2). The scoring range reflects the variability of some criteria and allows for a selection of a precise attribution, avoiding a black or white choice. By summing up the individual scores and reaching a score from +14 down to −9 points, the final RUCAM score indicates the causality level: score ≤ 0, excluded causality; 1–2, unlikely; 3–5, possible; 6–8, probable; ≥9, highly probable. To achieve high causality gradings, the proactive updated RUCAM should be used prospectively to ensure case data completeness [13]. Needless to say, many details are available in instructions on how best to apply the updated RUCAM and how to handle specific questions and conditions that may emerge during causality assessment [13,36,45]. To provide clear results, drug trials in DILI must use cases based on evaluation using the updated RUCAM to determine the grade of probability [13] and to ensure exclusion of cases due to nondrug alternative causes [38].

### 3.5. Randomized Controlled Trials

Randomized controlled trials (RCTs) are the gold standard for evaluation of therapy effectiveness in any clinical setting [46]. They represent prospective studies that measure efficacy of a new treatment and are applicable to drug treatment in DILI. RCT publications of drug treatment in DILI should follow the principles of CONSORT (Consolidated Standards of Reporting Trials) guidelines to improve the reporting of parallel-group randomized controlled trials [47]. With regrets, many publications on drug treatment efficiency in patients with DILI do not follow these prospective recommendations, thereby invalidating the results.

## 4. Hepatic Drug Handling

For initiating new study protocols regarding treatment of DILI by drugs, a look at details about how the liver handles drugs entering the organ may be helpful [48]. The liver is exposed to high concentrations of drugs, which are taken up orally and absorbed by the gut before they reach the liver via the venous portal system, ready for their uptake [49]. Drug concentrations in the hepatocytes depend on the relative speed of drug uptake, metabolism, and excretion [50,51,52].

### 4.1. Hepatocellular Drug Uptake

There is both passive drug diffusion from the blood and active drug influx via transporters such as NTCP (Na+-taurocholate cotransporting polypeptide), OCT (organic cation transporter), and OATP (organic anion transporting polypeptide). These processes are localized in the sinusoidal plasma membrane of the hepatocyte [50].

### 4.2. Hepatic Drug Metabolism

Drug biotransformation proceeds in the liver cell by metabolizing enzymes such as microsomal CYP isoforms [50,51] or non-CYP pathways like flavin-containing monooxygenase (FMO), monoamine oxidase (MAO), alcohol dehydrogenase (ADH), acetaldehyde dehydrogenase (ALDH), and aldehyde oxidase (AO) [50,52]. All these metabolic pathways are grouped as phase I reactions involving oxidation, reduction, or hydrolysis [50,51,52]. Subsequent pathways occur via conjugating enzymes and are grouped as phase II reactions [50]. Among these are UDP-glycosyltransferase (UGT), glutathione S-transferase (GST), sulfotransferase (SULT), and N-acetyltransferase (NAT) [50,52].

### 4.3. Elimination of Drugs and Their Metabolites

Classified as phase III, the elimination of the parent drug or its metabolites occurs preferentially via the bile canalicular pole of the plasma membrane of the hepatocyte by drug efflux mechanisms through transporters like bile salt export pump (BSEP), breast cancer resistance protein (BCRP), multidrug resistance protein (MDR), and multidrug resistance-associated protein (MRP) [50]. Several hundred drugs can induce DILI [16,53], which makes it difficult to assign for each drug reaction an individual mechanism of liver injury [54,55,56,57,58,59,60,61,62,63]. Additional challenges remain for defining a specific therapy that best fits for each DILI caused by any individual drug.

## 5. Basics of Molecular and Mechanistic Toxicology in DILI

The top drugs causing RUCAM-based DILI worldwide have been assessed as to whether they were metabolized through CYP isoforms [62]. Literature provided high-quality data on clinical features of DILI [1,2,3,4,5,6,7,8,9,10,16,17,18,19,20,21,22,23,24,25,26,27,28,39,64]. However, although most of the clinical features are well established, a variety of mechanistic steps remain unresolved in this complex disease. These molecular mechanisms include drugs with variable chemical structures, variabilities of clinical features and genetics of patients at risk, the multiplicity of non-parenchymal cells, in addition to the hepatocytes, exposed to the drugs entering the liver following intestinal absorption, and multiple immune cell types [62].

### 5.1. Liver Immune System

Compelling evidence exists that for most idiosyncratic DILI cases, the hepatic immune system plays a prominent pathogenetic role [61,62,63]. Briefly, the injury appears to be mediated mostly by CD8 T cells of the adaptive immune system, which requires prior activation of the innate immune system. Early steps in this process likely involve activation of antigen-presenting cells by molecules such as danger-associated molecular pattern molecules (DAMPs) [59]. Support for an involvement of the immune system in idiosyncratic DILI is provided by autoimmune parameters in the blood of patients, clinical features, liver histology, and in some cases, with human leucocyte antigen (HLA) genotypes [61,62,63]. Therefore, drugs or herbs offending the immune system are optional therapeuticals in DILI.

### 5.2. Hepatic Microsomal Cytochrome P450

The potential role of liver microsomal CYP, localized by definition in the smooth endoplasmic reticulum, visible at electronic microscopy, was analyzed with 48 top-ranking drugs involved in 3312 DILI cases, most of which had been assessed for causality with RUCAM and were published worldwide [65]. In at least 28/48 drugs (58.3%), clinical or experimental evidence exists that metabolism proceeds via CYPs, whereas for the remaining 20 drugs (41.7%), there were negative or missing results of metabolic participation of CYPs [62]. Among the various CYP isoforms, CYP 3A4 was the most frequent one (Table 2).

Listed are the top ranking 48 drugs implicated in causing 3312 idiosyncratic DILI cases with verified causality using RUCAM for 36/48 drugs #1–36 and without causality verification in the remaining 1748 drugs #37–48. The predominant CYP isoform involved in drug metabolism is listed. Abbreviations: CYP, cytochrome P450; DILI, drug-induced liver injury. The table is taken from an earlier open-access report [62].

**Figure 1:**
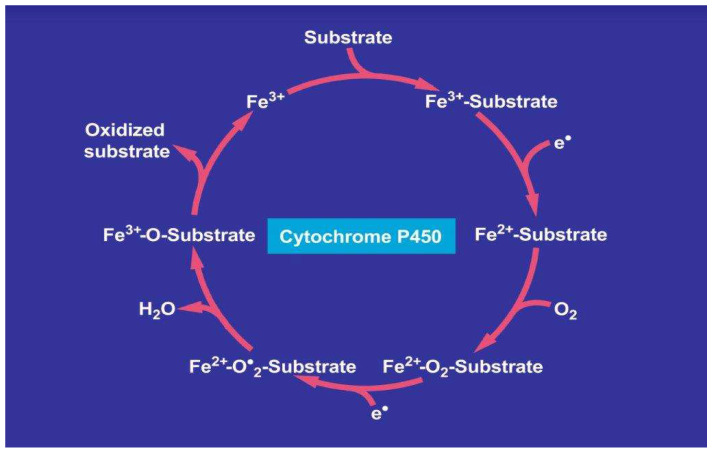
Catalytic CYP cycle of hepatic microsomal drug metabolism.

With respect to a potential therapy of patients with idiosyncratic DILI, the multiplicity of CYP isoforms (Table 2) impedes the recommendation of a single drug or herb for inhibiting all CYP isoforms to reduce toxicity. Under mechanistic aspects, drugs as listed above (Table 2) [62] and other substrates such as ethanol [66], phytochemicals [67], and 1,2-unsaturated pyrrolizidine alkaloids [68] may be oxidized via the CYP catalytic cycle. The tentative step-by-step approach is presented (Figure 1).

Drugs like other substrates enter the catalytic cytochrome P450 cycle as a substrate, shown on the top of the cycle. In the course of several mechanistic steps, the drug as a substrate leaves the cycle, providing it in its oxidized form as a new metabolite. Cytochrome P450 stands for its various isoforms. As a reminder, the term “P450” was used to describe a “pigment” with an absorption maximum at 450 nm with the ferrous-carbon monoxide complex of CYP in rat liver microsomes. The figure was adapted from a recent open-access report [68].

More specifically, the first electron is provided to CYP by NADPH + H^+^ via the NADPH CYP reductase, and the reduced form of CYP with Fe^2+^ is generated, which finally becomes oxidized again after being split off the oxidized substrate. CYP is then free again for the next substrate to be oxidized (Figure 1) [68]. Through the introduction of molecular oxygen, a multi-compound reactive complex emerges, facilitated by inclusion of another electron that commonly is provided through the NADPH CYP reductase or a similar but NADPH-independent reductase.

### 5.3. ROS and Oxidative Stress

Under normal physiological conditions, hepatic oxidative stress proceeds at a low level to generate sufficient ROS, required to sustain normal functions including drug oxidation. In idiosyncratic DILI, however, an overproduction of ROS can be assumed: part will be used for drug metabolism whereas the remaining ROS will injure the liver if antioxidant systems are exhausted [48,61,62,69,70,71,72]. The injury is triggered by various toxic intermediates such as superoxide O_2_^−•^, nitric oxide NO^•^, singlet oxygen ^1^O_2_, hydrogen peroxide H_2_O_2_, and peroxyl radical ROO^•^. These data are of importance under the aspect of potential therapeutic measures in replenishing the impaired antioxidant system in DILI.

### 5.4. Ferroptosis

Ferroptosis, also termed pyroptosis, was introduced as a new potential molecular and mechanistic concept in experimental intrinsic DILI by APAP [73,74] and various other liver diseases [74,75,76,77,78,79,80] such as alcoholic liver disease (ALD) [74], high-fat-diet-induced hepatic lipotoxicity [75], metabolic-associated fatty liver disease (MAFLD) [76], hereditary hemochromatosis [74], and liver injury patients with COVID-19 infections [79], perhaps attributable to DILI with verified diagnosis using RUCAM [17]. Similarly, pathogenetic involvement of ferroptosis was discussed in experimental toxic liver injury caused by the heavy metal nickel [80,81]. Using the experimental model of nickel (Ni) liver injury following NiCl_2_ administration, an increased iron content in the liver was found, associated with an upregulation of cyclooxygenase 2 (COX-2) protein and mRNA expression levels, downregulation of glutathione peroxidase 4 (GPX4), ferritin heavy chain 1 (FTH1), nuclear receptor coactivator 4 (NCOA4) protein, and mRNA expression levels. The conclusion was reached that nickel may cause hepatic injury through mitochondrial damage and ferroptosis [80]. Ferroptosis is defined as iron-dependent cell death, similar to glutamate-induced excitotoxicity but distinct from apoptosis, necrosis, and autophagy, and is triggered by inhibition of cystine uptake, whereby reduced cystine uptake leads to the production of lethal ROS [78]. Overall, the ferroptosis concept is highly complex and seems to be dependent on the Fenton reaction regarding the sequence of events with iron involvement in both [78,80,81]. First, the Fenton reaction with support of iron allows for the formation of radicals. Second, the radicals combine with polyunsaturated fatty acids (PUFA) to form lipid peroxides through pathways involving iron-containing enzymes, a process now called ferroptosis. Whether aspects of ferroptosis are relevant to clinical DILI and its therapy remains to be established.

### 5.5. Molecular Aspects of the Cholestatic DILI

The majority of idiosyncratic DILI cases present clinically as hepatocellular injury rather than as cholestatic injury [82,83]. As a consequence, pathogenetic steps leading to the cholestatic injury were rarely studied [61,62,63]. For instance, impairment of the hepatocellular bile salt export pumps (BSEP) was reported mostly from in vitro studies, but these results were not considered useful predictors of idiosyncratic DILI [63]. In addition, increased serum bile acid levels were less studied, but some details warrant mentioning [61]. In particular, the composition of bile acids is different in rodents as compared with humans regarding a higher percentage of the more polar and less toxic taurine conjugates in mice and more of the toxic glycine conjugates in humans [61,63]. This complicates the development of a valid rodent model to study the inhibition of BSEP or other bile acid transporters, which makes it more difficult to test the hypothesis that BSEP inhibition leads to cholestatic idiosyncratic DILI [61]. Thus, it remains to be established which mechanism triggers the cholestatic injury. Currently, it seems likely that transport systems and the concentration of a drug or its metabolites in the biliary system play an important role for pathogenesis and tentative treatment.

### 5.6. Gut Microbiome, Gut-Liver Axis, and DILI

There is compelling evidence from worldwide case reports on DILI assessed for causality using RUCAM that amoxicillin-clavulanate is on top of drugs causing this injury (Table 2) [62,65], as also correctly noted by others [84], and there is also much convincing data on disturbed diversity of gut bacteria following the use of antibiotics [84,85] including amoxicillin-clavulanate [84]. Putting these two lines of data together [62,65,84,85], it was postulated that changes of intestinal bacteria by antibiotics may produce not-yet-identified toxins, but diagnostic parameters such as serum endotoxins were not mentioned [84]. These unidentified toxins would enter the liver via the portal circulation and initiate, or at least contribute to, DILI development. Gut dysbiosis is known to cause a variety of liver diseases like ALD [86] or toxic liver injury by carbon tetrachloride, as examples [62]. Endotoxins syn lipopolysaccharides (LPS) are derived from the gut microbiome, enter the portal circulation through a leaky gut, and if not cleared by the liver, reach the systemic circulation where they can be quantified [87,88,89]. Published data on blood LPS were not available in patients with idiosyncratic DILI, although they were reported in animals exposed to APAP in overdose [62]. Antibiotics exaggerate idiosyncratic DILI likely through decreasing the good intestinal bacteria [90]. As it presently stands, LPS cannot assist in clarifying mechanistic steps in human idiosyncratic DILI nor can they provide proposals for therapy.

## 6. Initial Therapy of DILI by Drug Cessation

Cessation of all non-essential drugs is mandatory as soon as the idiosyncratic DILI is suspected [13,28,29,90,91]. This alone has a positive effect on LTs as shown in many reports on DILI with verified diagnosis based on the RUCAM [1,2,3,4,5,6,7,8,9]. In most cases, a complete resolution of the liver injury is achieved, while in a few others, a protracted course of LTs is observed. This eventually leads to chronic DILI or, in a worst-case scenario, to liver transplantation or death. Consequently, evidence-based pharmacotherapies are needed.

## 7. Action Principles of Potential Therapeuticals in DILI

A large number of pharmaceuticals, including conventional drugs, phytochemicals derived from herbal medicines, or other chemicals, are under discussion for potential use to treat patients with DILI. For DILI treatment, a list of selective, most commonly used potential pharmaceuticals or other chemicals is presented in alphabetical order with details of the tentative mechanisms of action (Table 3) [92,93,94,95,96,97,98,99,100,101,102,103,104,105,106,107,108,109].

Selected pharmaceuticals (conventional drugs and phytochemicals derived from herbal medicine) as possible candidates to treat patients with DILI are listed. Abbreviations: ABCB1, ATP binding Cassette Subfamily B member 1; APAP, N-Acetyl-p-aminophenol; BSEP, bile salt export pump; BSRP, brain-specific receptor-like protein C; CYP, cytochrome P450; DILI, drug-induced liver injury; GSH, glutathione; HSOS, hepatic sinusoidal obstruction syndrome; MRP2, multidrug resistance associated protein 2; ROS, reactive oxygen species; VDAC1, voltage-dependent anion selective channel 1.

Most of the listed pharmaceuticals were classified as antioxidants to be used against ROS in patients with drug-induced hepatocellular injury, some are applied for drug-induced cholestatic injury, while probiotics take care of the gut-liver axis, and a single medication group is used as anticoagulants in drug-induced HSOS (Table 3) [92,93,94,95,96,97,98,99,100,101,102,103,104,105,106,107,108,109]. Overall, mechanistic aspects of pharmaceuticals are interesting but the real world of DILI is more challenging, as treatment efficiency must be established by RCTs [46,47] with DILI cases assessed for causality using a robust diagnostic algorithm such as the updated RUCAM [13].

## 8. Published Reports of Drugs and Herbs Used for Therapy of DILI

In the past decade, much progress was recognizable in many segments of DILI research, with a focus on clinical characteristics [1,2,3,4,5,6,7,8,9,10,13,30,39,40,110], experimental studies on mechanistic steps [49,55,56,58,59,61,63,71,111], and drug development [25,26,27,28]. Despite these promising developments, DILI has been a treatment challenge nowadays as it was in the past, considering problems of previous therapeutic approaches as well as recent novel therapies [112]. There is much concern that the clinical management of DILI is, to a large extent, insufficiently studied and poorly documented. Shortcomings related to therapeutical DILI management were multiple and observed at different levels as reported in several publications [112,113,114], including scarcity of RCTs [114]. For instance, only eight RCTs were conducted in management of DILI or related acute liver failure: bicyclol (two studies, thereof one study using RUCAM-based cases), magnesium isoglycyrrhizinate (two studies, thereof one study using RUCAM-based cases), Silymarin (two studies), traditional Chinese medicines (two studies), and N-acetylcysteine (two studies) [114]. Critically among the eight RCTs, inclusion criteria of DILI were variable due to the inclusion of cases with ALT < five times the ULN, the lack of a robust causality assessment in most studies, and the multiplicity of drugs causing DILI not allowing the recommendation of a single treatment for DILI by a specific drug. There were additional challenges of studies dealing with therapy aspects in DILI (Table 4) [112,113,114]. 

Selected issues published in connection with studies on therapy in DILI. Abbreviations: DILI, drug-induced liver injury; RUCAM, Roussel Uclaf Causality Assessment method; ULN, upper limit of normal range.

Consensus exists that overall scientific evidence for use of specific medications in idiosyncratic DILI is weak, because well-powered and conducted RCTs are lacking [115]. It is obvious that confounding variables currently govern the issue of evidence-based recommendation of pharmacotherapy in cases of idiosyncratic DILI (Table 4) [112,113,114]. Considering the background information on mechanistic aspects of DILI (Table 3) and the outline on the limited study quality (Table 4), it expected that recommendations for drug therapy in DILI have commonly been published with caution [112,113,114]. Nonetheless, NAC is unquestionably the first-choice treatment of intrinsic APAP DILI, based on empirical efficiency but not on data derived from RCTs deemed not to be required any more. Despite basic uncertainties, GCs may be used empirically in selected patients with idiosyncratic DILI exhibiting autoimmune features or caused by immune checkpoint inhibitors (ICIs), while some patients with cholestatic DILI may benefit from UDCA use. Shortcomings are mostly related to both poor RCT quality and lack of causality verification. Already in 2007, the quality of reported RCTs addressing the issue of TCM efficiency was considered poor, based on an analysis of trial results published from 1999 to 2004 [116]. This study identified 37,252 Chinese language articles in TCM journals published in mainland China. Clinical trials were recognized in 26,263/37,252 articles, corresponding to 70.5% [116,117]. Among these 26,263 clinical trials, 7422 were initially identified as RCTs, equivalent to 28.3%, but of the 7422 trials, only 1329 (17.9%) were truly randomized [116]. Some important methodological components of the RCTs were incompletely reported, such as sample size calculation (reported in 1.1% of RCTs), randomization sequence (7.8%), allocation concealment (0.3%), implementation of the random allocation sequence (0%), and intension to treat analysis (0%). All reports were searched according to guidelines of the Cochrane Centre, and a comprehensive quality assessment of each RCT was completed using a modified version of the CONSORT checklist [116]. Although publications of TCM trials were abundant [116], their scientific quality is limited [117].

Some pharmaceuticals have been used to protect from upcoming DILI such as those caused by antituberculosis drugs; respective studies were not included in this paper but had been discussed in a recent careful analysis [114]. It is of note that many pharmaceuticals were used empirically in clinical settings to treat patients with idiosyncratic DILI and had been recommended in review articles or even in consensus statements, ignoring confounders and biases attributed to published study results. Despite shortcomings, there are a few examples of medications advocated for in DILI due to specific causative drugs. Some of the commonly considered medications below are discussed in alphabetical order.

### 8.1. Anticoagulants

Anticoagulants are the medicines of choice to treat patients with HSOS (Table 3), whether it is caused by drugs such as sirolimus, gemtuzumab, cyclophosphamide, or oxaliplatin, leading to drug-induced liver vascular injury [42,118,119], due to hematopoietic cell transplantation (HCT) [120,121,122], or following use of plants containing 1,2-unsaturated PAs [68,123,124]. HSOS is characterized among many other features by platelet aggravation and adhesion, damage of the endothelial cells, and the risk of occlusion of the hepatic sinusoids [68,118]. Approved by the FDA to treat HSOS caused by HCT, defibrotide is under discussion for use in HSOS by other causatives as this drug is a plasminogen inhibitor and thereby protects from blood clotting [121,122,124].

### 8.2. Antioxidants (General)

In addition to the listed antioxidants (Table 3), many plants with their antioxidant phytochemicals are candidates to be used for treating patients with idiosyncratic DILI [125,126]. For instance, a list of plants with the antioxidant flavonoids is available, whereby dihydroxy B-ring substituted flavonoids have a great potential to inhibit the production of ROS and reduce the levels of ROS once they are formed [125]. Other plants with antioxidant properties include *Alpinia zerumbet* [127,128,129], the fungus *Cordyceps militaris* [130,131], *Andropogon virginicus* [132], or *Coriza sativa* (rice) with its momilactones [133,134], but none of these herbs have been tested so far for their efficacy in DILI. Interestingly, plant antioxidants are generated under the influence of biotic or abiotic plant stress [135]. Biotic plant stress is caused by pathogen attacks of other living organisms like insects, larger grazing animals, parasites, bacteria, viruses, and fungi. Abiotic stress, on the other hand, is triggered by environmental attacks from unusual UV radiation, draft, wounding, or soil contamination by salts or heavy metals. At the molecular level, plant stress leads to antioxidant production triggered by oxidative stress. So far and with few exemptions, only a few herbal TCMs with antioxidant activities have successfully been tested in patients with DILI, but they may be more useful as preventing measures in patients under a drug therapy against tuberculosis [114].

### 8.3. Bicyclol

Bicyclol, a synthetic drug preventing hypoxic liver injury in animal models (Table 3) [94], improved serum ALT activities in two studies from China [114,136,137]. However, results were confounded as liver adaptation cases were included with serum ALT activities < five times of the ULN [136,137], and only one study used DILI cases with RUCAM scores of 6 and above equal to a probable or highly probable causality grading [137].

### 8.4. Cholestyramine

Cholestyramine as monotherapy or combined with antihistamines given orally may help relief pruritus in cholestatic DILI (Table 3) [28,138]. It may bind bile acids as well as some drugs, leading to an interruption of the entero-hepatic circulation [138]. There were no studies of efficacy regarding liver injury improvement.

### 8.5. Clausenamide

Clausenamide (CLA), a phytochemical with an alkaloid structure, was initially isolated from *Clausena lansium* and is now in its synthetic form, (+)-CLA, the most active initiator of hepatic GSH production as compared with its enantiomers (Table 3) [96]. It alleviates experimental intrinsic APAP liver injury, triggered by the ferroptosis mechanism, by reducing lipid peroxidation and increasing glutathione peroxidase activity [139]. It is no question that ferroptosis is a hot topic related to liver injury and discussed in various reports [73,74,75,76,77,78,79,80,81,82,83,84,85,86,87,88,89,90,91], as is CLA [96,139]. Studies of CLA in experimental APAP liver injury are interesting under scientific aspects but not necessarily relevant for human APAP hepatotoxicity because, for this entity, well-established therapeutic modalities are available using NAC as an antidote, which needs no update [103]. Whether ferroptosis is mechanistically involved in idiosyncratic DILI and CLA is a treatment option require further studies to answer open questions.

### 8.6. Glucocorticoids

Glucocorticoids (GCs) with a step-down therapy to suppress excessive inflammatory processes and immunological responses (Table 3) have widely been used empirically in patients with severe DILI. Treatment is by oral use of prednisone at a daily dose of 15 to 20 mg for 3 days by tapering the dose and seemingly without side effects [92]. The use of intravenous methylprednisolone in high doses is risky as it is associated with liver injury in selected patient cohorts and thus obsolete in DILI [39].

#### 8.6.1. DILI Related to Immune Checkpoint Inhibitors

Glucocorticoids (GCs) have correctly been recommended in selected patients with idiosyncratic DILI caused by immune checkpoint inhibitors (ICIs) [28], representing drugs approved by the US Federal Drug Administration (FDA) for the treatment of melanoma, certain subgroups of colorectal cancer, metastatic non-small cell lung cancer (NSCLC), hepatocellular carcinoma (HCC), urothelial carcinoma, bladder cancer, renal cell carcinoma (RCC), head and neck cancer, Hodgkin lymphoma, and Merkel cell carcinoma [140,141]. ICIs are immunomodulatory antibodies used to enhance the anticancer immune response, which is impaired in patients with advanced malignancy [141,142,143]. For checkpoint inhibition, two main targets are under discussion: (1) the programmed cell death receptor 1, PD-1, and the programmed cell death ligand 1, PDL-1, whereby PD-1 is involved in NK cell collapse, limiting the toxic power and production of cytokines, and (2), the cytotoxic T-lymphocytes-associated antigen 4, CTLA-4, which is implicated in the inhibition of IFN-γ assembly by natural killer (NK) cells [141]. Mechanistically, in the intestinal microbiome and at the hepatocellular level, ROS are likely involved in the ICI-related DILI [141]. Liver injury by ICIs was caused by drugs such as atezolizumab, avelumab, cemiplimab, durvalumab, ipilimumab, nivolumab, pembrolizumab, and tremelimumab [98,141,142]. They were responsible for the injury in up to 9% of the treated patients when taken as monotherapy [28,98], in 16% when used at a high dose [141], or up to 18% when applied in combination [98,141]. Grading systems are available for liver injury related to ICIs [113,144,145] such as the common terminology criteria for adverse events (CTCAE) [145]. When ALT is expressed as multiples of the ULN, liver injury grade 1 is confined to 1–2, grade 2 to 3–5, grade 3 to 5–20, and grade 4 to >20. Most of the patients had a liver injury grade of 2 or 3 [28], considering the CTCAE terminology [145].

As these patients with malignancy are mostly of elder age, likely with polymedication and concomitant diseases, the diagnosis of DILI by ICIs may be confounded by variables including alternative causes. This is why consensus exists that in all suspected ICI DILI cases, alternative causes should be excluded [28,98,113,140,141,144,146,147,148,149]. As an example, the most commonly identified alternative causes among 50 liver injury cases were progressive liver tumor metastases (56%), while other etiologies included malignant biliary obstruction (4%), non-hepatic diseases (9%), and other biliary obstructions or unknown [146]. Differentiation of ICI DILI from autoimmune diseases such as genuine autoimmune hepatitis (AIH), primary biliary cholangitis (PBC), primary sclerosing cholangitis (PSC), and their overlap syndromes has also been proposed [28].

RUCAM was used or proposed in many articles on DILI related to ICIs [113,140,141,144,146,147,148,149], with the understanding that DILI often is not DILI as the liver injury is due to non-drug causes [32,33,34,35]. For unknown reasons, RUCAM was not proposed or discussed for DILI cases due to ICIs, although was perfectly considered in many other DILI cases caused by various drugs [28]. RUCAM use as a tool of causality assessment remained also unconsidered by others [150,151]. More specifically, in a best practice paper on the ICI subject, RUCAM was ignored, making the best practice proposals less practicable [150]. RUCAM was also not mentioned in a safety paper, its conclusions therefore remaining vague [151]. The issue of RUCAM was, however, discussed in another publication [140] in reference to an excellent report on suspected 491 DILI cases caused by ICIs, which described that only 20 cases (28.6%) out of 491 cases were adjudicated as probably related to ICI treatment based on the RUCAM [146]. In fact, each patient with suspected DILI in connection with the use of ICIs should be assessed regarding causality for the suspected drug. This was recently done, for instance, in a perfect case report published with the title: acute liver failure following a single dose of atezolizumab, as assessed for causality using the updated RUCAM [149].

The use of GCs in selected patients with verified DILI related to ICIs is commonly accepted [28,148], based on details of a therapeutic algorithm [148] and provided that the ICI DILI diagnosis was ascertained with a probable or highly probable RUCAM causality grading. Accordingly, patients with serum ALT activities ≥ five times of the ULN (Table 1), corresponding to grade 3 of the CTCAE classification [145], were generally eligible for GC treatment after temporary discontinuation of the ICI treatment [28,148]. LTs should have been monitored over the next week, with the recognition that many patients would have improvement in the LTs with ICI cessation alone, and GC treatment should be initiated, empirically in the face of lacking RCTs, if LTs worsened despite ICI discontinuation [28,148]. There was concern if LTs did not improve despite ICI cessation and GC treatment. Under these conditions, the initial diagnosis of suspected DILI related to ICIs may have been missed and regarded as attributable to alternative causes not responsive to GC, commonly found in these patient cohorts with cancer disease. GC treatment in patients lacking a firm ICI DILI diagnosis may not only be ineffective but even worse, can be hazardous, because GC use is a risk factor for bacterial infections and sepsis that may require an antibiotic treatment, which in turn can disturb the intestinal microbiome and thus aggravate the DILI severity, as shown in cases of DILI assessed for causality using the updated RUCAM [84]. The aggravation of DILI severity by antibiotics is best explained by dysbiosis and barrier dysfunction that modify the disposal and action of other drugs [84,152]. Again, and as a reminder, it is recommended that all suspected DILI cases related to ICIs should undergo a robust causality assessment using the updated RUCAM, a recommendation regretfully not followed by a few reports [28,150,151].

#### 8.6.2. DILI by Common Drugs

GC treatment of patients with DILI is by no means supported by evidence because robust data derived from RCTs were not available [114]. Despite these uncertainties, GCs have empirically been used in unselected DILI cases due to various drugs [92,114,153,154,155,156]. In addition, therapy studies using GCs have provided controversial results, and consensus exists that a validated standard GC therapy for all DILI cases does not exist [92,153,154]. More specifically, problems of methodology included observational rather than RCT design, a comparison with historical controls, defining the best time starting GCs, the uncertainty whether GCs may even trigger acute liver failure, and a lack of data based on DILI cases assessed for causality with RUCAM. A step forward was noted with a recent GC therapy study from China, which provided an adequate protocol as the liver injury was clearly defined with ALT or AST activity levels ≥ 10 times of the ULN [155], although a threshold of ≥five times of the ULN would have been sufficient (Table 1) [15] to allow for inclusion of liver injury cases in the study cohort [13,15]. In addition, the updated RUCAM [13] was perfectly used to assess causality, also in line with recommendations of the Drug-induced Liver Injury (DILI) Study Group, Chinese Society of Hepatology (CSH), Chinese Medical Association (CMA) with their CSH guidelines for the diagnosis and treatment of drug-induced liver injury, Chinese researchers that proposed, for the evaluation of DILI cases, the application of the updated RUCAM [29], and only cases with a probable or highly probable RUCAM-based causality grading were included in the analysis [155]. The updated RUCAM excluded alternative causes, associated with confirming the chronicity of DILI. The therapeutic efficiency of GCs was classified as good with an improvement in laboratory data and liver histology and even better results when combined with glycyrrhizin, with no death or liver transplantation reported [155]. With regrets, the study had limitations due to the randomized open-label trial study design [155,156], an avoidable shortcoming easily preventable if a prospective RCT study design is used in an upcoming study. Thus, GCs should be carefully evaluated before application and used with great caution in DILI [156]. Current use of CSs remains a personal decision of the physician in each individual case *n* under special consideration of GC contraindications.

#### 8.6.3. Drug-Induced Autoimmune Hepatitis

Patients with drug-induced autoimmune hepatitis (DIAIH), a subgroup of conventional DILI but with additional clinical, laboratory, and liver histology features of autoimmunity, commonly benefit from empirical treatment with GCs [2,43,157,158,159]. As an example, the indication for the GC treatment in patients with RUCAM-based DIAIH was persistent or progressive serum ALT elevation despite withdrawal of the suspected causative drug or development of acute liver failure (AFL), with normalization of serum ALT activities after a median duration of 86 days [158]. In general, DIAIH as a diagnosis is easily accomplished if RUCAM scores [13] are combined with criteria scores for the diagnosis of genuine syn autoimmune hepatitis (AIH) [159,160,161]. High RUCAM causality scores associated with low diagnostic scores relevant for genuine, idiopathic AIH were in support of the DIAIH diagnosis [2,158,159,160]. There was a high number of studies on DIAIH that failed to use RUCAM to exclude alternative causes or missed gaining a high RUCAM based causality grading; they were thus of little clinical relevance as their results were not based on evidence, and they were also not further considered in this review. As it stands, DIAIH characteristics have well been described in RUCAM-based cases [2,157,159], especially if the updated RUCAM was applied [2,158,159]. This diagnostic approach also helped exclude a variety of alternative causes including cholangitis, cholelithiasis, primary biliary cholangitis, primary and secondary sclerosing cholangitis, other autoimmune diseases, alcoholic liver disease, non-alcoholic steatohepatitis, metabolic disorders, cardiac failure, and infections by cytomegalovirus (CMV), Epstein Barr virus (EBV), hepatitis A virus (HAV), hepatitis E virus (HEV), and human herpes virus (HHV). Many of these diseases have been listed as alternative causes in a separate table in the updated RUCAM paper [13]. Finally, in the absence of unequivocal diagnostic biomarkers, scores for DILI like RUCAM and for AIH can be used to distinguish both entities, DIAIH from genuine AIH [2,158,159].

### 8.7. Immune-Suppressants

Apart from GC, other immune-suppressants (Table 3) like mycophenolate mofetil (MMF), infliximab, and anti-thymocyte globulins have empirically been applied in patients with DILI, preferentially as second-line therapies following therapy failure with GCs [98]. The second-line immunosuppressants were used in a few patients only.

### 8.8. Iron Chelators

Baicalein, ciclopirox, desferoxamine, deferasirox, deferiprone, and dexrazoxane are iron chelators (Table 3) known to sequester iron from labile iron pool in cells [162]. They are candidates to counteract liver injury by iron via the ferroptosis mechanism [73,74,75,76,77,78,79,80,81]. Although the issue of iron and ferroptosis is fascinating and of potential value in clinical idiosyncratic DILI, there are currently no data to suggest the use of iron chelators in this type of liver injury [162,163,164]. Iron chelators have been protective in experimental intrinsic APAP liver injury [163], but this is not relevant for human intrinsic DILI by APAP because, for this intoxication, NAC is available as a highly efficient antidote without need of improvement [103].

### 8.9. L-Carnitine

Treatment with L-Carnitine (Table 3) applied intravenously was anecdotally described with positive effects in patients with DILI caused by valproate [165], but confirmation by RCTs is still lacking.

### 8.10. Magnesium Isoglycyrrhizinate

The efficiency of magnesium isoglycyrrhizinate (MgIG) (Table 3) was evaluated in a multicenter RCT phase II study, and the authors concluded that the trial provided preliminary evidence that MgIG is an effective treatment for acute DILI [166]. There is also the note that MgIG use in DILI could increase the efficiency of GC if administered together [155], raising the question whether GC plus MgIG could advance to a standard therapy [156]. Other trials using MgIG were summarized with a poor description of methodological design, with low stringent DILI diagnostic criteria while also including cases typical of liver adaptation rather than liver injury, with a short-term follow-up, and a lack of clinically meaningful endpoints [114]. Thus, the conclusion was reached that the validity of these results is compromised and should be interpreted cautiously.

### 8.11. N-Acetylcysteine

#### 8.11.1. APAP DILI

APAP poisoning may cause life-threatening intrinsic DILI including ALF [167,168,169,170] with ascertained diagnosis through causality assessment using RUCAM [171], and experimentally reproducible in animals showing prolonged alcohol consumption as a risk factor [172,173]. DILI by APAP is triggered by exhaustion of the hepatic content of glutathione, a powerful antioxidant commonly scavenging ROS and the reactive intermediate N-acetyl-p-benzo quinone imine (NAPQI) (Table 3), a toxic metabolite of APAP [103]. To replenish reduced hepatic glutathione levels, intravenous NAC was successfully applied to patients with ALF by APAP as shown with a substantial reduction of lethality compared with non-treated patients in a perfect RCT [174]. NAC is given intravenously according to the original recommendation: 150 mg/kg body weight in 200 mL 5% dextrose over 15 min, followed by 50 mg/kg in 500 mL 5% dextrose over 4 h, then 100 mg/kg in 1 L over 16 h, and considering other details as published [175]. Although various diagnostic biomarkers are under study to facilitate the diagnosis [176], a more practical diagnostic approach is required for patients with an acute single paracetamol overdose to classify the potential risk of toxicity [167,177]. Most commonly used is the classic Rumack–Matthew monogram of paracetamol concentration from the time of ingestion, applicable when the time of paracetamol ingestion is known and occurred within the preceding 24 h [177]. Plotting the time in hours versus paracetamol levels provides points on a so-called probable toxicity line: important values are paracetamol levels of 200 μg/mL at 4 h and 25 μg/mL at 16 h following acute intake [167]. Patients presenting higher paracetamol levels are at risk of severe liver injury as defined by ALT > 1000 U/L and require NAC treatment.

In cases of acute APAP intoxication, forced gastrointestinal lavage should be considered if APAP is likely still in the stomach [114]. The lavage method is also the approach commonly applied in intoxicated patients who ingested aliphatic halogenated hydrocarbons like carbon tetrachloride [178,179]. Since APAP is metabolically activated by CYP 2E1, the proposal has been made to inhibit this CYP isoform by intravenous application of cimetidine as soon as the intoxication is verified by actual exploration [171]. Whether charcoal depuration through application of carbo medicinalis via a nasogastric tube in patients intoxicated with APAP is effective remains to be established [112].

#### 8.11.2. Non-APAP DILI

In clinical practice, NAC is used anecdotally in patients with idiosyncratic DILI, as opposed to intrinsic DILI caused by APAP [174], but the efficacy of this approach is uncertain. An earlier analysis of RCTs and prospective cohort studies revealed that current available evidence is limited and does not allow for any firm recommendation to be made regarding the role of NAC in non-paracetamol DILI, calling for further research [180], which were conclusions confirmed recently [181]. Better designed RCTs are needed with the inclusion of DILI cases assessed for causality using the updated RUCAM.

### 8.12. Polyene Phosphatidylcholine

Polyene phosphatidylcholine (PPC) (Table 3) is used empirically for various liver diseases, including ALD worldwide but mostly in China, where PPC is applied also in patients with DILI [92]. Evidence of PPC efficiency is not mentioned due to lack of RCTs [114], or described as limited [92], and is otherwise poorly documented in English language publications and in adult populations [92]. However, in a recent study with RUCAM-based DILI cases, efficiency of PPC was found in a part of the patients when a propensity score matching a comparison method was applied [182]. It seems, therefore, that well-designed RCTs with RUCAM-based DILI cases are still needed to clarify the issue of efficiency.

### 8.13. Probiotics

Impairment of the gut microbiome following treatment with antibiotics may aggravate DILI, which led to the suggestion that probiotics (Table 3) or fecal microbiota transplantation may have a therapeutical potential [84]. However, data from RCTs of efficiency in patients with DILI are not available [114].

### 8.14. S-Adenosyl-Methionine

S-adenosyl-methionine (SAM) (Table 3) as a tentative treatment option of DILI is under discussion [92,112,183]. However, RCTs with robust data derived from RUCAM-based DILI cases were not available and SAM was thus not listed [114], not allowing currently for recommendation of its use in DILI.

### 8.15. Silymarin

Silymarin (Table 3) is one of the most studied medicines in DILI [114]. Data from RCTs are available but efficiency data were inconsistent [92,112]. Among five listed studies, three were retrospective, while the other two were open-label, randomized, or randomized double-blind clinical trials [92]. The use of silymarin seemed to be safe, but efficiency was either not verified or doubtful, and none of the DILI cases had been assessed for causality using RUCAM. In another otherwise comprehensive review, silymarin was not listed under the studies of efficiency with a certainty of evidence, and it was not discussed in the text [112]. An additional analysis reported on DILI patients treated with silymarin did not show clinical improvements in two management RCTs analyzed [114]. There was also the note that the Chinese Society of Hepatology (CSH) guidelines suggest that silymarin may be used to treat mild liver inflammation [29]. However, only three RCTs reported DILI according to its severity, which may limit the validity of the results, whereas partial support came from a trial with a small case number [114]. Since silymarin is widely used empirically in China, its high number of DILI patients should facilitate designing a prospective study protocol on silymarin in line with RCT rules with the use of RUCAM-based DILI cases.

### 8.16. Ursodeoxycholic Acid

Ursodeoxycholic acid (UDCA), a natural bile acid found in human bile (Table 3), was empirically used in patients with cholestatic DILI, based on anecdotal case reports describing improvement of LTs [30,39,92,109]. However, such positive effects as published in single case reports must be questioned because it might be ascribed to drug cessation alone in these individual cases. A lack of valid RCTs prevented inclusion of UDCA in a recent review article [114] and guidelines as thoroughly discussed [30,39,109]. Indeed, conclusions prevailed that the published studies do not allow a final recommendation on efficiency of UDCA in DILI [39,92,109]. Finally, international DILI guidelines did not recommend UDCA for treating patients with DILI or remained inconclusive and rather suggested a case-by-case decision [30,39,92,109].

## 9. Non-Pharmaceutical Treatment Approaches

Despite cessation of the suspected drug and initiation of pharmacotherapy, a small portion of patients with idiosyncratic and intrinsic DILI may proceed to ALF with coagulopathy and hepatic encephalopathy [6,92]. With imminent ALF, these patients must be transferred in time to special liver centers for further therapeutic decision regarding artificial liver support or liver transplantation.

### 9.1. Artificial Extracorporal Liver Support

Artificial extra-corporal liver support with a focus on mechanical, chemical, or biological devices may serve as a temporary replacement of impaired liver function, but efficiency remained under discussion [92].

### 9.2. Liver Transplantation

Patients with ALF due to DILI benefit from liver transplantation [92], provided the DILI diagnosis has been validly ascertained regarding RUCAM-based causality, an important point in the face of treatable alternative causes being confounding variables commonly observed among cohorts of suspected DILI [33,38]. Percentages of survival after liver transplantation are variable and often difficult to assess because in up to 68% of the overall ALF cases worldwide, the causes remained undetermined [184].

## 10. Proposals for the Future

Treatment modalities of intrinsic DILI caused by APAP intoxication are well established and in common use with a favorable outcome if NAC therapy is initiated shortly after the acute intoxication. Conversely, management of idiosyncratic DILI is challenging due to various shortcomings that impede the best therapy to be provided. To overcome these issues, the following steps should be considered: (1) there is an unmet need of more RCTs in line with CONSORT, which allows for a prospective study protocol with collection of all relevant case data, predefining patient criteria, and study endpoints; (2) the study cohort should include only cases of idiosyncratic DILI in connection with the use of a suspected drug; (3) each case considered for inclusion in the study must be assessed for causality for the drug under consideration, using a robust diagnostic algorithm such as the updated RUCAM, and only cases with a RUCAM-based causality grading of probable or highly probable are to be considered, excluding a priori cases with a causality grading of possible or less; (4) liver injury must be defined as serum ALT activity ≥ five times of the ULN and/or ALP ≥ two times of the ULN, in line with suggestions outlined in the report of the updated RUCAM and to rule cases with liver adaptation as evidenced by lower LT thresholds; (5) the study cohort must be homogenous by including only drugs as causatives and avoiding a mix with non-drugs such as herbal medicines or so-called herbal supplements; and (6) multi-center studies are preferred to ensure a high case number of the commonly rare DILI.

## 11. Conclusions

Based on empirical or theoretical evidence, discontinuation of the suspected drug(s) implicated in DILI is clinically the most common therapeutic approach. Additional therapy options with conventional drugs or phytochemicals in DILI are poorly documented. In particular, shortcomings of previous studies aiming to provide robust efficiency data included: (1) scarcity of good RCTs; (2) some liver injury study cohorts did not provide a list of offending drug(s), while others included conventional drugs used in recommended doses or overdoses, mixed with herbal medicines, alternative medications, ethyl alcohol, and alcohol surrogates; (3) heterogeneity of clinical presentation, disease severity, or toxicity grades; (4) ancillary analysis of liver injury severity; (5) lack of DILI definition, (6) divergent therapy efficacy results; (7) variability of efficacy criteria; (8) failure to consider and differentiate liver injury patterns as hepatocellular, cholestatic, or mixed; (9) medications not used alone but in combination with other medications; (10) a low case number of DILI study cohorts; (11) inclusion of cases with ALT values 2–5 times those of the ULN, thereby representing liver adaptation rather than real liver injury characteristics; (12) low or very low grades of certainty evidence reached in most studies; (13) retrospective rather than the preferred prospective study design; (14) bias with respect to selection (random sequence generation and allocation concealment), performance and detection of bias (blinding of participants, personnel, and outcome assessment); (15) a lack of data about causality assessment with only limited use of RUCAM; (16) partial-result publications of therapy benefits; (17) randomized, single blind rather than double blind study protocol; and (18) China as the preferred reporting country, as many treatment efficiency trials had their focus on traditional Chinese medicines (TCMs). Despite these conceptual flaws of efficiency data but in line with mechanistic considerations rather than resulting from evidence-based trials, selected patients with specific DILI features are commonly treated, for instance, with N-acetylcysteine (NAC), glucocorticoids, or ursodeoxycholic acids.

Under these conditions and as expected, international consensus papers are cautious and currently provide no or only vague recommendations and often propose treatment decisions on an individual basis tailored to injury specifics, which is generally not helpful for clinicians caring for patients with DILI, with the consequence that many patients were treated empirically. To overcome these problems, future studies of treatment options by drugs and phytochemicals in DILI should be confined to RCTs, which are privileged to have proactive, prospective, and complete case data collections with the need for clear criteria definitions of liver injury caused by conventional drugs, and ready for causality assessment using the updated RUCAM to provide valid data. 

## Figures and Tables

**Table 1:** Thresholds of ALT and ALP and typical features in patients with liver adaptation and liver injury.

Description	Thresholds of Liver Tests	Criteria and Characteristic Features
Liver adaptation	ALT ≤ 5 times of ULN and/or ALP ≤ 2 times of ULN	● Develops at low doses of a drug ● Presumably the majority of drugs have the potency of causing rare but clinically not apparent liver adaptation ● No signs of liver injury in histology ● Normalization or stabilization of liver tests is commonly observed whether the drug use is stopped or continued
Idiosyncratic liver injury	ALT ≥ 5 times of ULN and/or ALP ≥ 2 times of ULN	● Develops at low doses of a drug ● Signs of liver injury found in histology ● Cessation of drug use is mandatory and immediate ● Worsening if drug use is continued ● Most drugs cause idiosyncratic DILI ● Risk of acute liver failure
Intrinsic liver injury	ALT ≥ 5 times of ULN and/or ALP ≥ 2 times of ULN	● Develops with overdosed drugs ● Signs of liver injury found in histology ● Cessation of drug use is mandatory and immediate ● Caused by a few drugs ● Risk of acute liver failure

**Table 2:** Involvement of hepatic microsomal CYP isoforms in idiosyncratic DILI caused by various drugs as evaluated with RUCAM.

Drug	DILI Cases Evaluated with RUCAM (*n*)	Metabolized by CYP Isoform
1. Amoxicillin-clavulanate	333	-
2. Flucloxacilllin	130	CYP3A4
3. Atorvastatin	50	CYP3A4/5
4. Disulfiram	48	CYP2E1
5. Diclofenac	46	CYP2C8
6. Simvastatin	41	CYP3A4/5
7. Carbamazepine	38	CYP3A4/5
8. Ibuprofen	37	CYP2C8/9
9. Erythromycin	27	CYP3A4
10. Anabolic steroids	26	CYP2C19
11. Phenytoin	22	CYP2C9
12. Sulfamethoxazole/Trimethoprim	21	CYP2C9
13. Isoniazid	19	CYP2E1
14. Ticlopidine	19	CYP2C19
15. Azathioprine/ 6-Mercaptopurine	17	-
16. Contraceptives	17	CYP3A4
17. Flutamide	17	CYP1A2
18. Halothane	15	CYP2E1
19. Nimesulide	13	CYP2C9
20. Valproate	13	CYP2C9
21. Chlorpromazine	11	CYP2D6
22. Nitrofurantoin	11	-
23. Methotrexate	8	-
24. Rifampicin	7	-
25. Sulfazalazine	7	-
26. Pyrazinamide	6	-
27. Natriumaurothiolate	5	-
28. Sulindac	5	CYP1A2
29. Amiodarone	4	CYP3A4
30. Interferon beta	3	-
31. Propylthiouracil	2	CYP/NA
32. Allopurinol	1	-
33. Hydralazine	1	-
34. Infliximab	1	-
35. Interferon alpha/Peginterferon	1	-
36. Ketaconazole	1	-
37. Busulfan	0	-
38. Dantrolene	0	-
39. Didanosine	0	-
40. Efavirenz	0	CYP2B6
41. Floxuridine	0	-
42. Methyldopa	0	CYP/NA
43. Minocycline	0	-
44. Telithromycin	0	CYP3A4
45. Nevirapine	0	CYP3A4
46. Quinidine	0	CYP3A4
47. Sulfonamides	0	CYP/NA
48. Thioguanine	0	-

**Table 3:** Potential pharmaceuticals for treatment or prophylaxis of DILI.

Pharmaceutical	Tentative Mechanisms of Action in DILI	First Author
● **Anticoagulants**	Antithrombotic property such as in HSOS	Li 2022 [92]
● **Antioxidants (general)**	Antioxidants in general are chemicals with variable structures to help protect cells and subcellular organelles from oxidative injury caused by reactive oxygen species (ROS).	Ali 2020 [93]
● **Bicyclol**	Bicyclol is a synthetic drug that attenuates oxidative stress and endotoxins, partially via modulated expression of cytokines.	Yao 2009 [94]
● **Cholestyramine**	Cholestyramine used orally interrupts the enterohepatic circulation of bile acids.	Einarsson 1991 [95]
● **Clausenamide**	(+)-Clausenamide (CLA), a phytochemical initially isolated from leaves of *Clausena lansium* (Lour.) Skeels, increases in its synthetic form the hepatic cytosolic GSH content via stimulation of the key limiting enzyme γ-glutamylcysteine synthetase activity. Nowadays seen as a potential inhibitor of liver injury triggered through ferroptosis, CLA may help in DILI cases.	Wu 2006 [96]
● **Glucocorticoids (GCs)**	GCs suppress excessive inflammatory processes and immunological responses.	Ye 2022 [97]
● **Immuno**- **suppressants**	Immunosuppression of CD8^+^ T cell lobular and necrotic hepatitis	Corrigan 2019 [98]
● **Iron chelators**	The iron chelator deferoxamine and the ferroptosis inhibitor ferrostatin-1 alleviate ferroptosis in experimental acute APAP liver injury by protecting mitochondria via inhibiting voltage-dependent anion channel 1 (VDAC1) oligomerization by restoring hepatic ceramide and cardiolipin content.	Niu 2022 [99]
● **L-carnitine**	L-carnitine is an antioxidant with protective properties against lipid peroxidation, as evidenced by increased malondialdehyde concentrations due to oxidative stress in experimental APAP liver injury, whereby the positive effect can be attributed to an increase of hepatic GSH levels.	Yapar 2007 [100]
● **Magnesium isoglycyrrhizinate (MgIG)**	MgIG is a phytochemical extracted from licorice roots and known for its antioxidant, anti-inflammatory, and antiapoptotic characteristics. It inhibits oxidative stress and reduces the activities of superoxide dismutase and catalase, as well as levels of proinflammatory cytokines such as IL-1β, IL-6, and TNF-α. It also modifies the gut-liver axis by improving the gut microbial composition and intestinal barrier function.	Liu 2021 [101] Xia 2022 [102]
● **N-acetylcysteine (NAC)**	NAC is known for its strong antioxidant properties and its capacity to increase hepatic levels of glutathione, enabling some protection of liver injury by the reactive intermediate N-acetyl-p-benzo quinone imine (NAPQI) as the metabolite of APAP generated via CYP 2E1 and 1A2.	Ntamo 2021 [103]
● **Polyene phosphatidylcholine (PPC)**	PPC is a major component of membrane phospholipids, extract from soy, and rich in polyunsaturated fatty acids (PUFA), such as linoleic, linolenic, and oleic acids. It helps repair damaged membranes of the hepatocytes and relieve necroinflammation.	Fan 2022 [104]
● **Probiotics**	Probiotics such as *Lactobacillus reuteri*, *Lactobacillus rhamnosus*, *Bifidobacterium adolescens, Bacillus cereus*, *Akkermansia mucinophilia*, *Sacchoromyces boullardii*, and *Lactobacillus casei* help reshape the gut microbiota, reinforce gut barrier function, and modulate pathways to reduce cytokines and hepatic inflammation.	Chen 2021 [105]
● **S-Adenosyl-methionine (SAM)**	SAM participates in cellular reactions like transmethylation, transsulfuration, and aminopropylation; is the principal methyl donor in methyltransferase reactions; and the precursor for glutathione synthesis.	Noureddin 2020 [106]
● **Silymarin**	Silymarin is a phytochemical derived from the milk thistle, syn *Silybum marianum*, with polyphenols, flavonolignans, and flavonoids as its constituents. It inhibits ROS formation, functions as a scavenger of ROS once formed, increases the hepatic level of glutathione, decreases lipid peroxidation, stimulates the synthesis of proteins and phospholipids within the hepatocytes, and inhibits hepatic NF-κB activation.	Aghemo 2020 [107]
● **Ursodeoxycholic acid (UDCA)**	UCDA protects cholangiocytes against cytotoxic actions of hydrophobic bile acids, stimulates hepatobiliary secretion through enhanced expression of several transporter proteins like ABCB1, MRP2, and BSEP, and protects hepatocytes against apoptotic actions of bile acids.	Paumgartner 2002 [108] Parra-Landázury 2021 [109]

**Table 4:** Challenges of studies on therapy in DILI.

Challenges of Studies on Therapy Approaches in DILI	First Author
Some liver injury study cohorts did not provide a list of offending drug(s), while other cohorts included a mix of potential hepatotoxic compounds such as conventional drugs used at recommended doses, overdosed drugs like acetaminophen, herbal medicine, alternative medications, ethyl alcohol, and alcohol surrogates.	Benić 2022 [112]
Heterogeneity of clinical presentation, disease severity, or toxicity grades	Delire 2022 [113]
Ancillary analysis of liver injury severity	Niu 2020 [114]
Lack of DILI definition	Benić 2022 [112]
Divergent therapy efficacy results and variability of efficacy criteria	Niu 2020 [114]
Failure to consider and differentiate liver injury pattern as hepatocellular, cholestatic, or mixed	Delire 2022 [113] Niu 2020 [114]
Medications not used alone but in combination with other medications	Niu 2022 [114]
Low case number of DILI study cohorts	Benić 2022 [112] Niu 2020 [114]
Inclusion of cases with ALT values 2–5 times those of the ULN, thereby representing liver adaptation rather than real liver injury characteristics	Benić 2022 [112] Niu 2020 [114]
Low or very low grade of certainty evidence reached in most studies	Benić 2022 [112]
Retrospective rather than the preferred prospective study design	Benić 2022 [112]
Bias with respect to selection (random sequence generation and allocation concealment), performance and detection of bias (blinding of participants, personnel, and outcome assessment)	Benić 2022 [112] Niu 2020 [114]
Lack of data about causality assessment or with only limited use of RUCAM	Benić 2022 [112] Delire 2022 [113] Niu 2020 [114]
Randomized, single blind rather than double blind study protocol	Benić 2020 [112]
China as the preferred reporting country, as many treatment efficiency trials had their focus on traditional Chinese medicines (TCMs)	Benić 2022 [112] Niu 2020 [114]

## Data Availability

Data were derived from published reports.

## References

[1] Rodríguez A., García-García I., Soto L., Huertas A., Borobia A., González-Torbay A., Akatbach-Bousaid I., González-Muñoz M., Ramirez E. (2022). Utility of lymphoycyte transformation test for assisting updated Roussel Uclaf Causality Assessment Method in drug-induced liver injury: A case-control study. Front. Pharmacol..

[2] Abeles R.D., Foxton M., Khan S., Goldin R., Smith B., Thursz M.R., Verma S. (2020). Androgenic anabolic steroid-induced liver injury: Two case reports assessed for causality by the updated Roussel Uclaf Causality Assessment Method (RUCAM) score and a comprehensive review of the literature. BMJ Open Gastroenterol..

[3] Chen Y., Wang C., Yang H., Huang P., Shi J., Tong Y., Jiang J., Zhang X., Chen W., Xuan Z. (2021). Epidemiology of drug- and herb-induced liver injury assessed for causality using the updated RUCAM in two hospitals from China. BioMed Res. Int..

[4] Lunardelli M.M., Becker M.W., Ortiz G.X., Blatt C.R. (2022). Drug-induced liver injury causality assessment data from a crosssectional study In Brazil: A call for the use of updated RUCAM in hospital pharmacy. Rev. Bras. Farm. Hosp. Serv. Saude.

[5] Ye L., Feng Z., Huang L., Guo C., Wu X., He L., Tan W., Wang Y., Wu X., Hu B. (2021). Causality evaluation of drug-induced liver injury in newborns and children in the intensive care unit using the updated Roussel Uclaf Causality Assessment Method. Front. Pharmacol..

[6] Danjuma M.I.M., Almasri H., Alshokri S., Khir F.K., Elmalik A., Battikh N.G., Abdallah I.M.H.A., Elshafei M., Fatima H., Mohamed M.F.H. (2020). Avoidability of drug-induced liver injury (DILI) in an elderly hospital cohort with cases assessed for causality by the updated RUCAM score. BMC Geriatr..

[7] González-Muñoz M., Monserrat Villatoro J., Marín-Serrano E., Stewart S., Bardón Rivera B., Marín J., Martínez de Soto L., Seco Meseguer E., Ramírez E. (2020). A case report of a drug-induced liver injury (DILI) caused by multiple antidepressants with causality established by the updated Roussel Uclaf causality assessment method (RUCAM) and in vitro testing. Clin. Case Rep..

[8] Wurzburger R. (2022). A case of delayed hepatic injury associated with teriflunomide use as assessed for causality using the updated RUCAM. Case Rep. Hepatol..

[9] Shi X., Lao D., Xu Q., Li X., Lv Q. (2022). A case report of drug-induced liver injury after tigecycline administration: Histopathological evidence and a probable causality grading as assessed by the updated RUCAM diagnostic scale. BMC Infect. Dis..

[10] Plüß M., Tampe D., Schwörer H., Bremer S.C.B., Tampe B. (2022). Case report: Kinetics of human leukocyte antigen receptor HLA-DR during liver injury induced by potassium para-aminobenzoate as assessed for causality using the updated RUCAM. Front. Pharmacol..

[11] Danan G., Bénichou C. (1993). Causality assessment of adverse reactions to drugs–I. A novel method based on the conclusions of international consensus meetings: Application to drug-induced liver injuries. J. Clin. Epidemiol..

[12] Bénichou C., Danan G., Flahault A. (1993). Causality assessment of adverse reactions of drugs–II. An original model for validation of drug causality assessment methods: Case reports with positive rechallenge. J. Clin. Epidemiol..

[13] Danan G., Teschke R. (2016). RUCAM in drug and herb induced liver injury: The update. Int. J. Mol. Sci.

[14] Teschke R. (2020). DILI, HILI, RUCAM algorithm, and AI, the Artificial Intelligence: Provocative issues, progress, and proposals. Arch. Gastroenterol. Res..

[15] Teschke R., Danan G. (2021). Idiosyncratic drug-induced liver injury (DILI) and herb-induced liver injury (HILI): Diagnostic algorithm based on the quantitative Roussel Uclaf Causality Assessment Method (RUCAM). Diagnostics.

[16] Teschke R., Danan G. (2020). Worldwide use of RUCAM for causality assessment in 81,856 DILI and 14,029 HILI cases published 1993-mid 2020: A comprehensive analysis. Medicines.

[17] Teschke R., Méndez-Sánchez N., Eickhoff A. (2022). Liver injury in COVID-19 patients with drugs as causatives: A systematic review of 996 DILI cases published 2020/2021 based on RUCAM as causality assessment method. Int. J. Mol. Sci.

[18] Muhović D., Bojović J., Bulatović A., Vukčević B., Ratković M., Lazović R., Smolović B. (2020). First case of drug-induced liver injury associated with the use of tocilizumab in a patient with COVID-19. Liver. Int..

[19] Chen F., Chen W., Chen J., Xu D., Xie W., Wang X., Xie Y. (2021). Clinical features and risk factors of COVID-19-associated liver injury and function: A retrospective analysis of 830 cases. Ann. Hepatol..

[20] Delgado A., Stewart S., Urroz M., Rodríguez A., Borobia A.M., Akatbach-Bousaid I., González-Muñoz M., Ramírez E. (2021). Characterisation of drug-induced liver injury in patients with COVID-19 detected by a proactive pharmacovigilance program from laboratory signals. J. Clin. Med..

[21] Jothimani D., Vij M., Sanglodkar U., Patil V., Sachan D., Narasimhan G., Kaliamoorthy I., Rela M. (2021). Severe jaundice in a COVID-19 patient-virus or drug?. J. Clin. Exp. Hepatol..

[22] Kumar P., Kulkarni A., Sharma M., Raon P.N., Reddy D.N. (2021). Letter to the Editor. Favipiravir-induced liver injury in patients with coronavirus disease 2019. J. Clin. Transl. Hepatol..

[23] Yamazaki S., Suzuki T., Sayama M., Nakada T.A., Igari H., Ishii I. (2021). Suspected cholestatic liver injury induced by favipiravir in a patient with COVID-19. J. Infect Chemother..

[24] Naseralallah L.M., Aboujabal B.A., Geryo N.M., Al Boinin A., Al Hattab F., Akbar R., Umer W., Jabbar L.A., Danjuma M.I. (2022). The determination of causality of drug induced liver injury in patients with COVID-19 clinical syndrome. PLoS ONE.

[25] Sarges P., Steinberg J.M., Lewis J.H. (2016). Drug-induced liver injury: Highlights from a review of the 2015 literature. Drug Saf..

[26] Shahbaz O., Mahajan S., Lewis J.H. (2017). Highlights of drug- and herb-induced liver injury in the literature from 2016: How best to translate new information into clinical practice?. Exp. Opin. Drug Metab. Toxicol..

[27] Real M., Barnhill M.S., Higley C., Rosenberg J., Lewis J. (2019). Drug-induced liver injury: Highlights of the recent literature. Drug Saf..

[28] Clinton J.W., Kiparizoska S., Aggarwal S., Woo S., Davis W., Lewis J.H. (2021). Drug-induced liver injury: Highlights and controversies in the recent literature. Drug Saf..

[29] Yu Y.C., Mao Y.M., Chen C.W., Chen J.J., Chen J., Cong W.M., Ding Y., Duan Z.P., Fu Q.C., Guo X.Y. (2017). Drug-induced Liver Injury (DILI) Study Group, Chinese Society of Hepatology (CSH), Chinese Medical Association (CMA). CSH guidelines for the diagnosis and treatment of drug-induced liver injury. Hepatol. Int..

[30] Devarbhavi H., Aithal G., Treeprasertsuk S., Takikawa H., Mao Y., Shasthry S.M., Hamid S., Tan S.S., Philips C.A., George J. (2021). Drug-induced liver injury: Asia Pacific Association of Study of Liver consensus guidelines. Hepatol. Int..

[31] Aithal G.P., Watkins P.B., Andrade R.J., Larrey D., Molokhia M., Takikawa H., Hunt C.M., Wilke R.A., Avigan M., Kaplowitz N. (2011). Case definition and phenotype standardization in drug-induced liver injury. Clin. Pharmacol. Ther..

[32] Teschke R., Danan G. (2021). DILI cases in registries and databases: An analysis of quality. Int. J. Gastroenterol. Hepatol. Dis..

[33] Teschke R., Danan G. (2021). The LiverTox paradox-gaps between promised data and reality check. Diagnostics.

[34] Björnsson E.S. (2016). Hepatotoxicity by drugs: The most common implicated agents. Int. J. Mol. Sci..

[35] Björnsson E.S., Hoofnagle J.H. (2016). Categorization of drugs implicated in causing liver injury: Critical assessment based on published case reports. Hepatology.

[36] Danan G., Teschke R. (2019). Roussel Uclaf Causality Assessment Method for drug-induced liver injury. Front. Pharmacol..

[37] Danan G., Teschke R. (2022). Electronic RUCAM: Major pitfalls call for caution and proper validation. Hepatology.

[38] Teschke R., Danan G. (2018). Drug induced liver injury with analysis of alternative causes as confounding variables. Br. J. Clin. Pharmacol..

[39] Méndez-Sánchez N., Teschke R., Méndez-Sánchez N. (2022). Drug-induced liver injury by conventional drugs, using cases based on the Roussel Uclaf Causality Assessment Method. Comprehensive Guide to Hepatitis Advances.

[40] Ke L., Lu C., Shen R., Lu T., Ma B., Hua Y. (2020). Knowledge mapping of drug-induced liver injury: A scientometric investigation (2010–2019). Front. Pharmacol..

[41] Kolaric T.O., Nincevic V., Kuna L., Duspara K., Bojanic K., Vukadin S., Raguz-Lucic N., Wu G.Y., Smolic M. (2021). Drug-induced fatty liver disease: Pathogenesis and treatment. J. Clin. Transl. Hepatol..

[42] DeLeve L.D., McCuskey R.S., Wang X., Hu L., McCuskey M.K., Epstein R.B., Kanel G.C. (1999). Characterization of a reproducible rat model of hepatic veno-occlusive disease. Hepatology.

[43] Sebode M., Schulz L., Lohse A.W. (2017). “Autoimmune(-like)” drug and herb induced liver injury: New insights into molecular pathogenesis. Int. J. Mol. Sci..

[44] Teschke R., Danan G., Chen M., Yvonne Will Y. (2018). Causality assessment methods in drug-induced liver injury. Drug-induced Liver Toxicity.

[45] Danan G., Teschke R. (2018). Drug-induced liver injury: Why is the Roussel Uclaf Causality Assessment Method (RUCAM) still used 25 years after its launch?. Drug Saf..

[46] Hariton E., Locascio J.J. (2018). Randomised controlled trials–the gold standard for effectiveness research. BJOG.

[47] Moher D., Hopeell S., Schulz K.F., Montori V., Gøtzsche P.C., Devereaux P.J., Elbourne D., Egger M., Altman D.G. (2010). CONSORT 2010 explanation and elaboration: Updated guidelines for reporting parallel group randomised trials. J. Clin. Epi..

[48] Teschke R., Danan G. (2021). Idiosyncratic drug induced liver injury, cytochrome P450, metabolic risk factors, and lipophilicity: Highlights and controversies. Int. J. Mol. Sci..

[49] Jaeschke H., Gores G.J., Cederbaum A.I., Hinson J.A., Pessayre D., Lemasters J.J. (2002). Mechanisms of hepatotoxicity. Toxicol. Sci..

[50] Roth A.D., Lee M.Y. (2017). Idiosyncratic drug-induced liver injury (IDILI): Potential mechanisms and predictive assays. Biomed. Res. Int..

[51] Kalra B.S. (2007). Cytochrome P450 enzyme isoforms and their therapeutic implications: An update. Indian. J. Med. Sci..

[52] Foti R.S., Dalvie D.K. (2016). Cytochrome P450 and non-cytochrome P450 oxidative metabolism: Contributions to the pharmacokinetics, safety, and efficacy of xenobiotics. Drug Metab. Dispos..

[53] Teschke R. (2019). Idiosyncratic DILI: Analysis of 46,266 cases assessed for causality by RUCAM and published from 2014 to early 2019. Front. Pharmacol..

[54] Corsini A., Bortolini M. (2013). Drug-induced liver injury: The role of drug metabolism and transport. J. Clin. Pharmacol..

[55] Johansson I., Ingelman-Sundberg M. (2011). Genetic polymorphism and toxicology—With emphasis on cytochrome P450. Toxicol. Sci..

[56] Guengerich F.P. (2007). Mechanisms of cytochrome P450 substrate oxidation: MiniReview. J. Biochem. Mol. Toxicol..

[57] Tarantino G., Di Minno M.N., Capone D. (2009). Drug-induced liver injury: Is it somehow foreseeable?. World J. Gastroenterol..

[58] Uetrecht J. (2007). Idiosyncratic drug reactions: Current understanding. Annu. Rev. Pharmacol. Toxicol..

[59] Uetrecht J.P. (1999). New concepts in immunology relevant to idiosyncratic drug reaction: The “danger hypothesis” and innate immune system. Chem. Res. Toxicol..

[60] Teschke R., Danan G. (2020). Liver injury by drugs metabolized via cytochrome P450. J. Mod. Med. Chem..

[61] Uetrecht J. (2019). Mechanistic studies of idiosyncratic DILI: Clinical implications. In: Special issue: Clinical drug induced liver injury: Current diagnostic and mechanistic challenges, guest editors: Rolf Teschke, Gaby Danan, James, H. Lewis. Front. Pharmacol..

[62] Teschke R., Uetrecht J. (2021). Mechanism of idiosyncratic drug induced liver injury (DILI): Unresolved basic issues. In special issue: Unresolved basic issues in hepatology, guest editors Ralf Weiskirchen & Wolfgang Stremmel. Ann. Transl. Med..

[63] Jee A., Sernoskie S.C., Uetrecht J. (2021). Idiosyncratic drug-induced liver injury: Mechanistic and clinical challenges. Int. J. Mol. Sci..

[64] Díaz-Orozco L., Quiroz-Compean F., Aquino-Matus J., Teschke R., Méndez-Sánchez N. (2022). Severe DILI in a patient under polypharmacy including rosuvastatin: Diagnostic challenges and lessons from a case report assessed using the updated RUCAM algorithm. Int. J. Gastroenterol. Hepatol. Dis..

[65] Teschke R. (2018). Top-ranking drugs out of 3312 drug-induced liver injury cases evaluated by the Roussel Uclaf Causality Assessment Method. Expert. Opin. Drug Metab. Toxicol..

[66] Teschke R. (2018). Alcoholic liver disease: Alcohol metabolism, cascade of molecular mechanisms, cellular targets, and clinical aspects. Biomedicines.

[67] Brewer C.T., Chen T. (2017). Hepatotoxicity of herbal supplements mediated by modulation of cytochrome P450. Int. J. Mol. Sci..

[68] Teschke R., Vongdala N., Quan N.V., Quy T.N., Xuan T.D. (2021). Toxifying 1,2-unsaturated pyrrolizidine alkaloids causing human hepatic sinusoidal obstruction syndrome. Int. J. Mol. Sci..

[69] Mihajlovic M., Vinken M. (2022). Mitochondria as the target of hepatotoxicity and drug-induced liver injury: Mechanism and detection methods. Int. J. Mol. Sci..

[70] Studentova H., Volakova J., Spisarova M., Zemankova A., Aiglova K., Szotkowski T., Melichar B. (2022). Severe tyrosine-kinase inhibitor induced liver injury in metastatic renal cell carcinoma patients: Two case reports assessed for causality using the updated RUCAM and review of the literature. BMC Gaastroenterol..

[71] Jaeschke H., Ramachandran A. (2011). Reactive oxygen species in the normal and acutely injured liver. J. Hepatol..

[72] Cichoż-Lach H., Michalak A. (2014). Oxidative stress as a crucial factor in liver diseases. World J. Gastroenterol..

[73] Lőrincz T., Jemnitz K., Kardon T., Mandl J., Szarka A. (2015). Ferroptosis is involved in acetaminophen induced cell death. Pathol. Oncol. Res..

[74] Macías-Rodríguez R.U., Inzaugarat M.E., Ruiz-Margáin A., Nelson L.J., Trautwein C., Cubero F.J. (2020). Reclassifying hepatic cell death during liver damage: Ferroptosis-a novel form of non-apoptotic cell death?. Int. J. Mol. Sci..

[75] Jiang J.J., Zhang G.F., Zheng J.Y., Sun J.H., Ding S.B. (2022). Targeting mitochondrial ROS-mediated ferroptosis by quercetin allivates high-fat diet-induced hepatic lipotoxicity. Front. Pharmacol..

[76] Zhang H., Zhang E., Hu H. (2021). Role of ferroptosis in non-alcoholic fatty liver disease and its implications for therapeutic strategies. Biomedicines.

[77] Gan B. (2021). Mitochondrial regulation of ferroptosis. J. Cell Biol..

[78] Dixon S.J., Lemberg K.M., Lamprecht M.R., Skouta R., Zaitsev E.M., Gleason C.E., Patel D.N., Bauer A.J., Cantley A.M., Yang W.S. (2012). Ferroptosis: An iron-dependent form of nonapoptotic cell death. Cell.

[79] Chen Y., Xu Y., Zhang K., Shen L., Deng M. (2022). Ferroptosis in COVID-19-related liver injury: A potential mechanism and therapeutic target. Front. Cell Infect. Microbiol..

[80] Wei L., Zuo Z., Yang Z., Yin H., Yang Y., Fang J., Cui H., Du Z., Ouyang P., Chen X. (2022). Mitochondria damage and ferroptosis involved in Ni-induced hepatotoxicity in mice. Toxicology.

[81] Teschke R. (2022). Aluminum, Arsenic, Beryllium, Cadmium, Chromium, Cobalt, Copper, Iron, Lead, Mercury, Molybdenum, Nickel, Platinum, Thallium, Titanium, Vanadium, and Zinc: Molecular aspects in experimental liver injury. Int. J. Mol. Sci..

[82] Zhu Y., Niu M., Chen J., Zou Z., Ma Z., Liu S., Wang R., He T., Song H., Wang Z. (2016). Hepatobiliary and pancreatic: Comparison between Chinese herbal medicine and Western medicine-induced liver injury of 1985 patients. J. Gastroenterol. Hepatol..

[83] Jing J., Teschke R. (2018). Traditional Chinese medicine (TCM) and herb induced liver injury: Comparison with drug induced liver injury. J. Clin. Trans. Hepatol..

[84] Fu L.H., Qian Y.H., Shang Z., Sun X.H., Kong X.N., Gao Y.Q. (2022). Antibiotics enhancing drug-induced liver injury assessed for causality using Roussel Uclaf Causality Assessment ethod: Emerging role of gut microbiota dysbiosis. Front. Med..

[85] Lange K., Buerger M., Stallmach A., Bruns T. (2016). Effects of antibiotics on gut microbiota. Dig. Dis..

[86] Teschke R., Zhu Y. (2019). Opinion: Intestinal microbiome, endotoxins, cytochrome P450 2E1, and the gut-liver axis in alcoholic liver disease. EC Gastroenterol. Dig. Syst..

[87] Cani P.D. (2018). Human gut microbiome: Hopes, threats and promises. Gut.

[88] Nolan J.P. (2010). The role of intestinal endotoxin in liver injury: A long and evolving history. Hepatology.

[89] Zhang J., Wang R. (2018). Gut microbiota modulates drug pharmacokinetics. Drug Metab. Rev..

[90] Becker M.W., Lunardelli M.J.M., Tovo C.V., Blatt C.R. (2019). Drug and herb-induced liver injury: A critical review of Brazilian cases with proposals for the improvement of causality assessment using RUCAM. Ann. Hepatol..

[91] Gerbes A.L. (2021). Drug-induced liver injury (DILI): A major challenge. Drug Res..

[92] Li M., Luo Q., Tao Y., Sun X., Liu C. (2022). Pharmacotherapies for drug-induced liver injury: A current literature review. Front. Pharmacol..

[93] Ali S.S., Ahsan H., Zia M.K., Siddiqui T., Khan F.H. (2020). Understanding oxidants and antioxidants: Classical team with new players. J. Food Biochem..

[94] Yao X.M., Chen H., Li Y. (2009). Protective effect of bicyclol on liver injury induced by hepatic warm ischemia/reperfusion in rats. Hepatol. Res..

[95] Einarsson K., Ericsson S., Ewert S., Reihnér E., Rudling MStåhlberg D., Angelin B. (1991). Bile acid sequestrants: Mechanisms of action on bile acid and cholesterol metabolism. Eur. J. Clin. Pharmacol..

[96] Wu Y.Q., Liu L.D., Wei H.L., Liu G.T. (2006). Different effects of nine clausenamide ennatiomers on liver glutathione biosynthesis and glutathione S-transferase activity in mice. Acta Pharmacol. Sin..

[97] Ye C., Li W., Li L., Zhang K. (2022). Glucocorticoid treatment strategies in liver failure. Front. Immunol..

[98] Corrigan M., Haydon G., Thompson F., Rajoriya N., Peplow C.L., Hubscher S.G., Steven N., Hirschfield G.M., Armstrong M.J. (2019). Infliximab for the treatment of refractory immune-related hepatitis secondary to checkpoint inhibitors: A case report. J. Hepatol. Rep..

[99] Niu B., Lei X., Xu Q., Ju Y., Xu D., Mao L., Li J., Zheng Y., Sun N., Zhang X. (2022). Protecting mitochondria via inhibiting VDAC1 oligomerization alleviates ferroptosis in acetaminophen-induced acute liver injury. Cell Biol. Toxicol..

[100] Yapar K., Kart A., Karapehlivan M., Atakisi O., Tunca R., Erginsoy S., Citil M. (2007). Hepatoprotective effect of L-carnitine against acute acetaminophen toxicity in mice. Exp. Toxicol. Pathol..

[101] Liu M., Zheng B., Liu P., Zhang J., Chu X., Dong C., Shi J., Liang Y., Chu L., Liu Y. (2021). Exploration of the hepatoprotective effect and mechanism of magnesium isoglycyrrhizinate in mice with arsenic trioxide-induced acute liver injury. Mol. Med. Rep..

[102] Xia Y., Shi H., Qian C., Han H., Lu K., Tao R., Gu R., Zhao Y., Wei Z., Lu Y. (2022). Modulation of gut microbiotica by magnesium isoglycyrrhizinate mediates enhancment of intestinal barrier function and amelioration of methotrexate-induced liver injury. Front. Immunol..

[103] Ntamo Y., Ziqubu K., Chellan N., Nkambule B.B., Nyambuya T.M., Mazibuko-Mbeje S.E., Gabuza K.B., Marcheggiani F., Tiano L., Dludla P.V. (2021). Drug-induced liver injury: Clinical evidence of N-acetyl cysteine protective effects. Oxidat. Med. Cell. Longevity.

[104] Fan J.G., Li Y., Yu Z., Luo X.X., Zheng P., Hao X., Wang Z.Y., Gao F., Zhang G.Q., Feng W.Y. (2022). Effectiveness and economic evaluation of polyene phosphatidyl choline in patients with liver diseases based on real-world research. Front. Pharmacol..

[105] Chen T., Li R., Chen P. (2021). Gut microbiota and chemical-induced acute liver injury. Front. Phys..

[106] Noureddin M., Sander-Struckmeier S., Mato J.M. (2020). Early treatment efficacy of S-adenosylmethionine in patients with intrahepatic cholestasis: A systematic review. World J. Hepatol..

[107] Aghemo A., Alekseeva O.P., Angelico F., Bakulin I.G., Bakulina N.V., Bordin D., Bueverov A.O., Drapkina O.M., Gillessen A., Kagarmanova E.M. (2022). Role of silymarin as antioxidant in clinical management of chronic liver diseases: A narrative review. Ann. Med..

[108] Paumgartner G., Breuer U. (2002). Ursodeoxycholic acid in cholestatic liver disease: Mechanisms of action and therapeutic use revisited. Hepatology.

[109] Parra-Landázury N.M., Córdova-Gallardo J., Méndez-Sánchez N. (2021). Drug induced liver injury: Is there an indication for ursodeoxycholic acid use?. J. Mod. Med. Chem..

[110] Devarbhavi H., Joseph T., Kumar N.S., Rathi C., Thomas V., Singh S.P., Sawant P., Goel A., Eapen C.E., Rai P. (2021). The Indian Network of Drug-Induced Liver Injury: Etiology, clinical features, outcome and prognostic markers in 1288 patients. J. Clin. Exp. Hepatol..

[111] Meunier L., Larrey D. (2019). Drug-Induced Liver Injury: Biomarkers, requirements, candidates, and validation. Front. Pharmacol..

[112] Benić M.S., Nežić L., Vujić-Aleksić V., Mititelu-Tartau L. (2022). Novel therapies for the treatment of drug-induced liver injury: A systematic review. Front. Pharmacol..

[113] Delire B., De Martin E., Meunier L., Larrey D., Horsmans Y. (2022). Immunotherapy and gene therapy: New challenges in the diagnosis and management of drug-induced liver injury. Front. Pharmacol..

[114] Niu H., Sanabria-Cabrera J., Alvarez-Alvarez I., Robles-Diaz M., Stankevičiūtė S., Aithal G.P., Björnsson E.S., Andrade R.J., MLucena M.I. (2021). Prevention and management of idiosyncratic drug-induced liver injury: Systematic review and meta-analysis of randomised clinical trials. Pharmacol. Res..

[115] Garcia-Cortes M., Robles-Diaz M., Stephens C., Ortega-Alonso A., Lucena M.I., Andrade R.J. (2020). Drug induced liver injury: An update. Arch. Toxicol..

[116] Wang G., Mao B., Xiong Z.Y., Fan T., Chen X.D., Wang L., Liu G.J., Liu J., Guo J., Chang J. (2007). CONSORT Group for Traditional Chinese Medicine. The quality of reporting of randomized controlled trials of traditional Chinese Medicine: A survey of 13 randomly selected journals from mainland China. Clin. Ther..

[117] Teschke R., Wolff A., Frenzel C., Eickhoff A., Schulze J. (2015). Herbal traditional Chinese medicine and its evidence base in gastrointestinal disorders. World J. Gastroenterol..

[118] Zhu C., Ren X., Liu D., Zhang C. (2021). Oxaliplatin-induced hepatic sinusoidal obstruction syndrome. Toxicology.

[119] Zhou S.N., Feng D.N., Zhang N., Sun Y.L., Li Y.W., Zhou X., Yang J., Liu Z., Liu H. (2021). Hepatic sinusoidal obstruction syndrome due to tacrolimus in a liver-transplantation recipient. Gastroenterol. Rep..

[120] Dignan F.L., Wynn R.F., Hadzic N., Karani J., Quaglia A., Pagliuca A., Veys P., Potter M.N., Haemato-oncology Task Force of British Committee for Standards in Haematology, British Society for Blood and Marrow Transplantation (2013). BCSH/BSBMT guideline: Diagnosis and management of veno-occlusive disease (sinusoidal obstruction syndrome) following haematopoietic stem cell transplantation. Br. J. Haematol..

[121] Liu Z.L., Wang Y., Liu Y.L., Zhang J. (2021). Application of defibrotide in hepatic sinusoidal obstruction syndrome induced by hematopoietic stem cell transplantation]. Zhonghua Gan Zang Bing Za Zhi.

[122] Chalandon Y., Mamez A.C., Giannotti F., Beau verd Y., Dantin C., Mahne E., Mappoura M., Bernard F., de Ramon Ortiz C., Stephan C. (2022). Defibrotide Shows Efficacy in the prevention of sinusoidal obstruction syndrome after allogeneic hematopoietic stem cell transplantation: A retrospective study. Transplant. Cell Ther..

[123] Gao H., Ruan J.Q., Chen J., Li N., Ke C.Q., Ye Y., Lin G., Wang J.Y. (2015). Blood pyrrole-protein adducts as a diagnostic and prognostic index in pyrrolizidine alkaloid-hepatic sinusoidal obstruction syndrome. Drug Des. Dev. Ther..

[124] Zhuge Y., Liu Y., Xie W., Zou X., Xu J., Wang J., Chinese Society of Gastroenterology Committee of Hepatobiliary Disease (2019). Expert consensus on the clinical management of pyrrolizidine alkaloid-induced hepatic sinusoidal obstruction syndrome. J. Gastroenterol. Hepatol..

[125] Agati G., Azzarello E., Pollastri S., Tattini M. (2012). Flavonoids as antioxidants in plants: Location and functional significance. Plant. Sci..

[126] Teschke R., Xuan T.D. (2020). Active nature based ingredients for drug discovery with pivotal role of clinical efficacy: Review and prospective. J. Mod. Med. Chem..

[127] Xuan T.D., Teschke R. (2015). Dihydro-5,6-dehydrokavain (DDK) from Alpinia zerumbet: Its isolation, synthesis, and characterization. Molecules.

[128] Teschke R., Xuan T.D. (2018). Viewpoint: A contributory role of Shell ginger (Alpinia zerumbet) for human longevity of Okinawa in Japan?. Nutrients.

[129] Teschke R., Xuan T.D., Victor R.P., Vinood B.P. (2020). Herbs including shell ginger, antioxidant profiles, aging, and longevity in Okinawa, Japan: A critical analysis of current concepts. Aging: Oxidative Stress and Dietary Antioxidants.

[130] Jedrejko K.J., Lazur J., Muszynska B. (2021). Cordyceps militaris: An overview of its chemical constituents in relation to biological activity. Foods.

[131] Quy T.N., Xuan T.D., Andriana Y., Tran H.D., Khanh T.D., Teschke R. (2019). Cordycepin Isolated from Cordyceps militaris: Its newly discovered herbicidal property and potential plant-based novel alternative to Glyphosate. Molecules.

[132] Anh L.H., Quan N.V., Lam V.Q., Iuchi Y., Takami A., Teschke R., Xuan T.D. (2021). Antioxidant, anti-tyrosinase, anti-α-amylase, and cytotoxic potentials of the invasive weed Andropogon virginicus. Plants.

[133] Minh T.N., Xuan T.D., Ahmad A., Elzaawely A.A., Teschke R., Van T.M. (2018). Momilactones A and B: Optimization of yields from isolation and purification. Separations.

[134] Quan N.V., Tran H.D., Xuan T.D., Ahmad A., Dat T.D., Khanh T.D., Teschke R. (2019). Momilactones A and B are α-amylase and α-glucosidase inhibitors. Molecules.

[135] Teschke R., Zhu Y., Jing J. (2020). Herb induced liver injury (HILI) in the Asian region and current role of RUCAM for causality assessment in 11,160 published cases: Analysis and outlook. J. Clin. Transl. Hepatol..

[136] Tang R.L. (2013). Analysis of the efficacy of bicyclol tablets in the treatment of liver injury caused by the anti-tuberculosis drug. Xinxueguanbing Fangzhi Zhishi.

[137] Naqiong W., Liansheng W., Zhanying H., Yuanlin G., Chenggang Z., Ying G., Qian D., Dongchen L., Yanjun Z., Jianjun L. (2017). A multicenter and randomized controlled trial of bicyclol in the treatment of statin-induced liver injury. Clin. Res..

[138] Stine J.G., Lewis J.H. (2016). Current and future directions in the treatment and prevention of drug-induced liver injury: A systematic review. Expert Rev. Gastroenterol. Hepatol..

[139] Wang M., Liu C.Y., Wang T., Yu H.M., Ouyang S.H., Wu Y.P., Gong H.B., Ma X.H., Jiao G.L., Fu L.L. (2020). (+)-Clausenamide protects against drug-induced liver injury by inhibiting hepatocyte ferroptosis. Cell Death Dis..

[140] Da Cunha T., Wu G.Y., Yazin H. (2022). Immunotherapy-induced hepatotoxicity: A review. J. Clin. Trans. Hepatol.

[141] Malnick S.D.H., Abdullah A., Neuman M.G. (2021). Checkpoint Inhibitors and hepatotoxicity. Biomedicines.

[142] Cho Y.A., Han J.M., Kang S.Y., Kim D.C., Youn Y.J., Choi K.H., Gwak S.H. (2021). Analysis of risk factors for hepatotoxicity induced by immune checkpoint inhibitors. J. Immunother..

[143] Sharma P., Allison J.P. (2015). The future of immune checkpoint therapy. Science.

[144] Remash D., Prince D.S., McKenzie C., Strasser S.I., Kao S., Liu K. (2021). Devika Remash, David S Prince, Catriona McKenzie, Simone, I. Strasser, Ken Liu. Immune checkpoint inhibitor-related hepatotoxicity: A review. World J. Gastroenterol..

[145] Colevas A.D., Setser A. (2004). The NCI Common Terminology Criteria for Adverse Events (CTCAE) v 3.0 is the new standard for oncology clinical trials. J. Clin. Oncol..

[146] Tsung I., Dolan R., Lao C.D., Fecher L., Riggenbach K., Yeboah-Korang A., Fontana R.J. (2019). Liver injury is most commonly due to hepatic metastases rather than drug hepatotoxicity during pembrolizumab immunotherapy. Aliment. Pharmacol. Ther..

[147] Swanson L.A., Kassab I., Tsung I., Schneider B.J., Fontana R.J. Liver injury during durvalumab-based immunotherapy is associated with poorer patient survival: A retrospective ananlysis. Front. Pharmacol..

[148] De Martin E., Michot J.M., Papouin B., Champiat S., Mateus C., Lambotte O., Roche B., Antonini T.M., Coilly A., Laghouati S. (2018). Characterization of liver injury induced by cancer immunotherapy using immune checkpoint inhibitors. J. Hepatol..

[149] Tzadok R., Levy S., Aouizerate J., Shibolet O. (2022). Acute liver failure following a single dose of atezolizumab, as assessed for causality using the updated RUCAM. Case Rep. Gastrointest Med..

[150] Regev A., Avigan M.I., Kiazand A., Vierling J.M., Lewis J.H., Omokaro S.O., Di Bisceglie A.M., Fontana R.J., Bonkovsky H.L., Freston J.W. (2020). Best practices for detection, assessment and management of suspected immune-mediated liver injury caused by immune checkpoint inhibitors during drug development. J. Autoimmun..

[151] Weber J.S., Hodi F.S., Wolchok J.D., Topalian S.L., Schadendorf D., Larkin J., Sznol M., Long G.V., Li H., Waxman I.M. (2017). Safety profile of nivolumab monotherapy: A pooled analysis of patients with advanced melanoma. J. Clin. Oncol..

[152] Weersma R.K., Zhernakova A., Fu J. (2020). Interaction between drugs and the gut microbiome. Gut.

[153] Hu P.F., Xie W.F. (2019). Corticosteroid therapy in drug-induced liver injury: Pros and cons. J. Dig. Dis..

[154] Björnsson E.S., Vucic V., Stirnimann G., Robles-Díaz M. (2022). Role of corticosteroids in drug-induced liver injury. A systematic review. Front. Pharmacol..

[155] Wang J.B., Huang A., Wang Y., Ji D., Liang Q.S., Zhao J., Zhou G., Liu S., Niu M., Sun Y. (2022). Corticosteroid plus glycyrrhizin therapy for chronic drug- or herb-induced liver injury achieves biochemical and histological improvements: A randomised open-label trial. Aliment. Pharmacol. Ther..

[156] Teschke Teschke R., Eickhoff A. (2022). Chronic DILI and HILI-corticosteroid plus glycyrrhizin as standard therapy?. Aliment. Pharmacol. Ther..

[157] Tan C.K., Ho D., Wang L.M., Kumar R. (2022). Drug-induced autoimmune hepatitis: A minireview. World J. Gastroenterol..

[158] Weber S., Benesic A., Rotter I., Gerbes A.L. (2019). Early ALT response to corticosteroid treatment distinguishes idiosyncratic drug-induced liver injury from autoimmune hepatitis. Liver. Int..

[159] Tsang L., Fadia M., Chitturi S. (2016). A time of pause and reflect: When a patient with autoimmune hepatitis stops responding to corticosteroids. Case Rep. Gastrointest. Med..

[160] Alvarez F., Berg P.A., Bianchi F.B., Bianchi L., Burroughs A.K., Cancado E.L., Chapman R.W., Cooksley W.G., Czaja A.J., Desmet V.J. (1999). International Autoimmune Hepatitis Group Report: Review of criteria for diagnosis of autoimmune hepatitis. J. Hepatol..

[161] Hennes E.M., Zeniya M., Czaja A.J., Parés A., Dalekos G.N., Krawitt E.L., Bittencourt P.L., Porta G., Boberg K.M., Hofer H. (2008). Simplified criteria for the diagnosis of autoimmune hepatitis. Hepatology.

[162] Chen X., Yu C., Kang R., Tang D. (2020). Iron metabolism in ferroptosis. Front. Cell Dev. Biol..

[163] Sakaida I., Kayano K., Wasaki S., Nagatomi A., Matsumura Y., Okita K. (1995). Protection against acetaminophen-induced liver injury in vivo by an iron chelator, deferoxamine. Scand. J. Gastroenterol..

[164] Casale M., Picariello S., Corvino F., Cerasari G., Scianguetta S., Rossi F., Persico M., Perrotta S. (2018). Life-threatening drug-induced liver injury in a patient with β-thalassemia major and severe iron overload on polypharmacy. Hemoglobin.

[165] Bohan T.P., Helton E., McDonald I., König S., Gazitt S., Sugimoto T., Scheffner D., Cusmano L., Li S., Koch G. (2001). Effect of L-carnitine treatment for valproate-induced hepatotoxicity. Neurology.

[166] Wang Y., Wang Z., Gao M., Zhong H., Chen C., Yao Y., Zhang Z., Zhang X., Li F., Zhang J. (2019). Efficacy and safety of magnesium isoglycyrrhizinate injection in patients with acute drug-induced liver injury: A phase II trial. Liver Int..

[167] Yoon E., Babar A., Choudhary M., Kutner M., Pyrsopoulos N. (2016). Acetaminophen-induced hepatotoxicity: A comprehensive update. J. Clin. Transl. Hepatol..

[168] Pholmoo N., Bunchorntavakul C. (2019). Characteristics and outcomes of acetaminophen overdose and hepatotoxicity in Thailand. J. Clin. Transl. Hepatol..

[169] Bunchorntavakul C., Reddy K.R. (2018). Acetaminophen (APAP or N-Acetyl-p-Aminophenol) and acute liver failure. Clin. Liver Dis..

[170] Ramachandran A., Jaeschke H. (2018). Acetaminophen toxicity: Novel insights into mechanisms and future perspectives. Gene Expr..

[171] Teschke R., Nikolaos T.P. (2020). Acetaminophen syn. paracetamol: Acute liver injury and acute on chronic liver failure with case analysis and causality assessment using RUCAM. Liver Failure.

[172] Teschke R., Stutz G., Strohmeyer G. (1979). Increased paracetamol-induced hepatotoxicity after chronic alcohol consumption. Biochem. Biophys. Res. Commun..

[173] Teschke R., Zhu Y. (2018). Paracetamol (acetaminophen), alcohol, and liver injury: Biomarkers, clinical issues, and experimental aspects. SL Pharmacol. Toxicol..

[174] Keays R., Harrison P.M., Wendon J.A., Forbes A., Grove C., Alexander G.J.M., Williams R. (1991). Intravenous acetylcysteine in paracetamol induced fulminant hepatic failure: A prospective controlled trial. Br. Med. J..

[175] Prescott L.F., Illingworth R.N., Critchley J.A.J.H., Stewart M.J., Adam R.D., Proudfoot A.T. (1979). Intravenous N-acetylcysteine; the treatment of choice for paracetamol poisoning. Br. Med. J..

[176] Teschke R., Eickhoff A., Brown A.C., Neuman M.G., Schulze J. (2020). Diagnostic biomarkers in liver injury by drugs, herbs, and alcohol: Tricky dilemma after EMA correctly and officially retracted Letter of Support. Int. J. Mol. Sci..

[177] Rumack B.H., Matthew H. (1975). Acetaminophen poisoning and toxicity. Pediatrics.

[178] Teschke R. (2018). Aliphatic halogenated hydrocarbons: Liver injury in 60 patients. J. Clin. Trans. Hepatol..

[179] Teschke R. (2018). Liver injury by carbon tetrachloride intoxication in 16 patients treated with forced ventilation to accelerate toxin removal via the lungs: A clinical report. Toxics.

[180] Chughlay M.F., Kramer N., Spearman C.W., Werfalli M., Cohen K. (2016). N-acetylcysteine for non-paracetamol drug-induced liver injury: A systematic review. Br. J. Clin. Pharmacol..

[181] Sanabria-Cabrera J., Tabbai S., Niu H., Alvarez-Alvarez I., Licata A., Björnsson E., Andrade R.J., Lucena M.I. (2022). N-acetylcysteine for the management of non-acetaminophen drug-induced liver injury in adults: A systematic review. Front. Pharmacol..

[182] Lei X., Zhang J., Xu Q., Li J., Qian Y., Zhang J., Liu L., Zhong W., Wang Y., Han X. (2021). Exploring the efficacy and safety of polyene phosphatidylcholine for treatment of drug-induced liver injury using the Roussel Uclaf causality assessment method: A propensity score matching comparison. J. Int. Med. Res..

[183] Santini D., Vincenzi B., Massacesi C., Picardi A., Vespasiani-Gentilucci U., Esposito V., Liuzzi G., La Cesa A., Rocci L., Marcucci F. (2003). S-adenosylmethionine (AdoMet) supplementation for treatment of chemotherapy-induced liver injury. Anticancer Res..

[184] Teschke R., Schmidt M. (2020). Controversy on a newly published case of assumed acute liver failure one day after kava use: Issues of confounders, causality, and an undetermined cause. J. Mod. Med. Chem..

